# A third dose of inactivated vaccine augments the potency, breadth, and duration of anamnestic responses against SARS-CoV-2

**DOI:** 10.1093/procel/pwae033

**Published:** 2024-05-27

**Authors:** Zijing Jia, Kang Wang, Minxiang Xie, Jiajing Wu, Yaling Hu, Yunjiao Zhou, Ayijiang Yisimayi, Wangjun Fu, Lei Wang, Pan Liu, Kaiyue Fan, Ruihong Chen, Lin Wang, Jing Li, Yao Wang, Xiaoqin Ge, Qianqian Zhang, Jianbo Wu, Nan Wang, Wei Wu, Yidan Gao, Jingyun Miao, Yinan Jiang, Lili Qin, Ling Zhu, Weijin Huang, Yanjun Zhang, Huan Zhang, Baisheng Li, Qiang Gao, Xiaoliang Sunney Xie, Youchun Wang, Yunlong Cao, Qiao Wang, Xiangxi Wang

**Affiliations:** CAS Key Laboratory of Infection and Immunity, National Laboratory of Macromolecules, Institute of Biophysics, Chinese Academy of Sciences, Beijing 100101, China; CAS Key Laboratory of Infection and Immunity, National Laboratory of Macromolecules, Institute of Biophysics, Chinese Academy of Sciences, Beijing 100101, China; Key Laboratory of Medical Molecular Virology (MOE/NHC/CAMS), Shanghai Institute of Infectious Disease and Biosecurity, Shanghai Frontiers Science Center of Pathogenic Microorganisms and Infection, School of Basic Medical Sciences, Shanghai Medical College, Fudan University, Shanghai 200032, China; Division of HIV/AIDS and Sex-Transmitted Virus Vaccines, Institute for Biological Product Control, National Institutes for Food and Drug Control (NIFDC), Beijing 102629, China; Sinovac Biotech Ltd., Beijing 100085, China; Fundamental Research Center, Shanghai Yangzhi Rehabilitation Hospital (Shanghai Sunshine Rehabilitation Center), School of Medicine, Tongji University, Shanghai 201619, China; Beijing Advanced Innovation Center for Genomics (ICG), Biomedical Pioneering Innovation Center (BIOPIC), School of Life Science, Peking University, Beijing 100091, China; CAS Key Laboratory of Infection and Immunity, National Laboratory of Macromolecules, Institute of Biophysics, Chinese Academy of Sciences, Beijing 100101, China; CAS Key Laboratory of Infection and Immunity, National Laboratory of Macromolecules, Institute of Biophysics, Chinese Academy of Sciences, Beijing 100101, China; CAS Key Laboratory of Infection and Immunity, National Laboratory of Macromolecules, Institute of Biophysics, Chinese Academy of Sciences, Beijing 100101, China; CAS Key Laboratory of Infection and Immunity, National Laboratory of Macromolecules, Institute of Biophysics, Chinese Academy of Sciences, Beijing 100101, China; CAS Key Laboratory of Infection and Immunity, National Laboratory of Macromolecules, Institute of Biophysics, Chinese Academy of Sciences, Beijing 100101, China; Guangzhou Laboratory, Guangzhou 510320, China; Sinovac Biotech Ltd., Beijing 100085, China; Sinovac Biotech Ltd., Beijing 100085, China; Beijing Advanced Innovation Center for Genomics (ICG), Biomedical Pioneering Innovation Center (BIOPIC), School of Life Science, Peking University, Beijing 100091, China; Sinovac Biotech Ltd., Beijing 100085, China; Key Laboratory of Medical Molecular Virology (MOE/NHC/CAMS), Shanghai Institute of Infectious Disease and Biosecurity, Shanghai Frontiers Science Center of Pathogenic Microorganisms and Infection, School of Basic Medical Sciences, Shanghai Medical College, Fudan University, Shanghai 200032, China; Key Laboratory of Medical Molecular Virology (MOE/NHC/CAMS), Shanghai Institute of Infectious Disease and Biosecurity, Shanghai Frontiers Science Center of Pathogenic Microorganisms and Infection, School of Basic Medical Sciences, Shanghai Medical College, Fudan University, Shanghai 200032, China; CAS Key Laboratory of Infection and Immunity, National Laboratory of Macromolecules, Institute of Biophysics, Chinese Academy of Sciences, Beijing 100101, China; Key Laboratory of Medical Molecular Virology (MOE/NHC/CAMS), Shanghai Institute of Infectious Disease and Biosecurity, Shanghai Frontiers Science Center of Pathogenic Microorganisms and Infection, School of Basic Medical Sciences, Shanghai Medical College, Fudan University, Shanghai 200032, China; Key Laboratory of Medical Molecular Virology (MOE/NHC/CAMS), Shanghai Institute of Infectious Disease and Biosecurity, Shanghai Frontiers Science Center of Pathogenic Microorganisms and Infection, School of Basic Medical Sciences, Shanghai Medical College, Fudan University, Shanghai 200032, China; Acrobiosystems Inc., Beijing 102600, China; Acrobiosystems Inc., Beijing 102600, China; Acrobiosystems Inc., Beijing 102600, China; CAS Key Laboratory of Infection and Immunity, National Laboratory of Macromolecules, Institute of Biophysics, Chinese Academy of Sciences, Beijing 100101, China; Sinovac Biotech Ltd., Beijing 100085, China; Department of Microbiology, Zhejiang Provincial Center for Disease Control and Prevention, Hangzhou 310051, China; Guangdong Provincial Center for Disease Control and Prevention, Guangzhou 511430, China; Guangdong Provincial Center for Disease Control and Prevention, Guangzhou 511430, China; Sinovac Biotech Ltd., Beijing 100085, China; Beijing Advanced Innovation Center for Genomics (ICG), Biomedical Pioneering Innovation Center (BIOPIC), School of Life Science, Peking University, Beijing 100091, China; Changping Laboratory, Beijing 102206, China; Division of HIV/AIDS and Sex-Transmitted Virus Vaccines, Institute for Biological Product Control, National Institutes for Food and Drug Control (NIFDC), Beijing 102629, China; Changping Laboratory, Beijing 102206, China; Beijing Advanced Innovation Center for Genomics (ICG), Biomedical Pioneering Innovation Center (BIOPIC), School of Life Science, Peking University, Beijing 100091, China; Changping Laboratory, Beijing 102206, China; Key Laboratory of Medical Molecular Virology (MOE/NHC/CAMS), Shanghai Institute of Infectious Disease and Biosecurity, Shanghai Frontiers Science Center of Pathogenic Microorganisms and Infection, School of Basic Medical Sciences, Shanghai Medical College, Fudan University, Shanghai 200032, China; CAS Key Laboratory of Infection and Immunity, National Laboratory of Macromolecules, Institute of Biophysics, Chinese Academy of Sciences, Beijing 100101, China; Guangzhou Laboratory, Guangzhou 510320, China; Changping Laboratory, Beijing 102206, China

**Dear Editor,**

The ongoing coronavirus disease 2019 (COVID-19) pandemic caused by severe acute respiratory syndrome coronavirus-2 (SARS-CoV-2) has lasted for more than four years, resulting in an unprecedented global public health crisis. Progress in halting this pandemic seems slow due to the emergence of variants of concern, such as the B.1.1.7 (Alpha), B.1.351 (Beta), P.1 (Gamma, also known as B.1.1.28.1), B.1.617.2 (Delta), and B.1.1.529 (Omicron), that appear to be high transmissible and more resistant to neutralizing antibodies (Wang et al., 2021g). New variants are thought to be responsible for re-infections (Hacisuleyman et al., 2021). A general decrease in immune protection against SARS-CoV-2 variants within 6–12 months after the primary infection or vaccination is also observed (Widge et al., 2021). However, not much is known about the immunogenic features of such a booster dose of a COVID-19 vaccine. In addition, there are large gaps in our understanding ofcorrelating immunogenic findings from surrogate endpoints to gauge vaccine efficacy.

The CoronaVac, a 3-dose β-propiolactone-inactivated vaccine against COVID-19, has been approved for emergency use by the World Health Organization (Gao et al., 2020). To evaluate immune features, we recruited 22 COVID-19 convalescents, 6 healthy participants (SARS-CoV-2 negative, confirmed by RT-PCR), and 38 volunteers who received either 2 or 3 doses of the Coronavac vaccine for blood donation. None of the volunteers recruited for vaccination was infected by SARS-CoV-2 before the study. Blood samples from convalescents and vaccinees collected 1.3 months after infection and the indicated times after vaccination were used in this study, respectively, to compare humoral immune responses elicited against circulating SARS-CoV-2 variants.

Neutralizing antibodies (NAbs) are a major correlate of protection for many viruses, including SARS-CoV-2. Neutralizing activity of plasma samples from 66 participants was measured against WT, B.1.351, P.1 and B.1.617.2 using live SARS-CoV-2 and VSV-pseudoviruses with the S from WT, B.1.1.7, P.1 variants and SARS-CoV (Fig. 1). The geometric mean half-maximal neutralizing titers (GMT NT_50_) against live SARS-CoV-2 in plasma obtained from convalescents and from vaccinees suggest an approximately 60% higher neutralizing activity against WT after 3-dose inoculation when compared with 2-dose administration, and 20% higher than those from convalescents (Fig. 1A). Interestingly, for the samples from the convalescents, 2-dose and 3-dose vaccinees, neutralizing titers against B.1.351 were, on average, 7.7-fold, 5.7-fold and 3.0-fold reduced, respectively, compared with WT (Fig. 1A). Similarly, fold decreases in neutralization ID_50_ titers against P.1 and B.1.617.2 for the three cohorts were 5.3, 4.3 and 3.1, and 5.3, 3.7 and 2.3, respectively (Fig. 1A). Overall, plasma of the 3-dose vaccinees displayed minimal reduction in neutralization titers against several authentic VOCs compared to the convalescents and 2-dose vaccinees (Fig. 1A). In line with the results of live SARS-CoV-2 neutralization assay, the mean fold decrease in the neutralization of B.1.1.7 relative to the WT was 2.8-fold for convalescents, 2.2-fold for 2-dose vaccinees and 1.7-fold for 3-dose vaccinees by using pseudo-typed viruses (Fig. 1B). Similarly, plasma from convalescents, 2-dose and 3-dose vaccinees exhibited a 4.5-fold, 2.9-fold and 2.4-fold reduction, in NAb titers against P.1, respectively, when compared to the WT (Fig. 1B). These results reveal that a third-dose boost of inactivated vaccine leads to enhanced neutralizing breadth to SARS-CoV-2 variants when compared to convalescent plasma.

**Figure 1.**
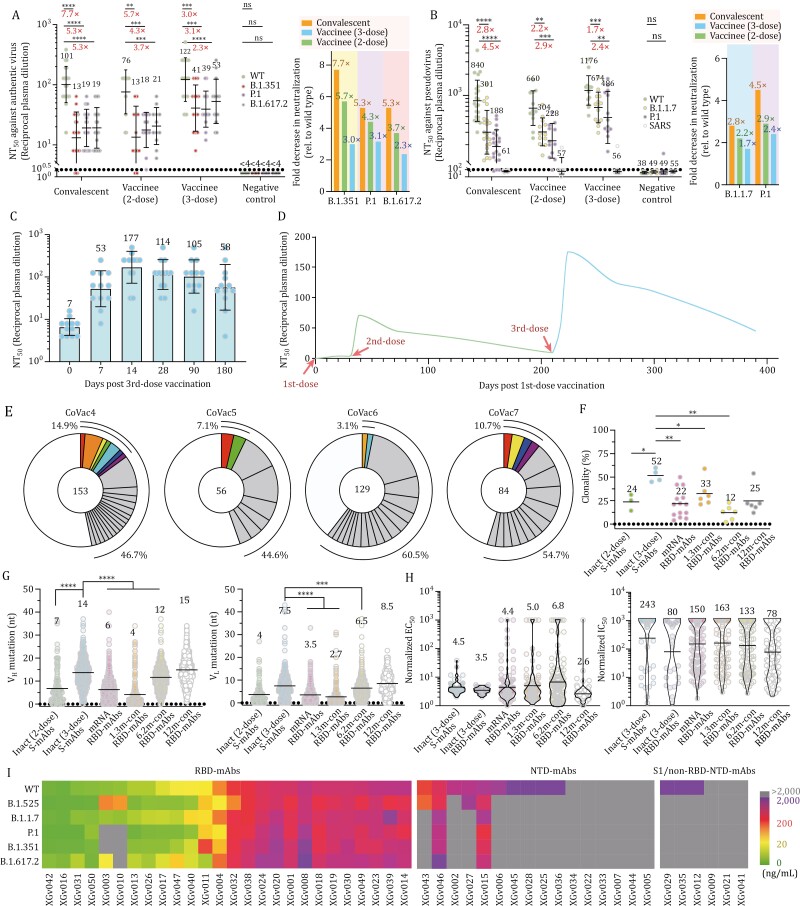
Landscape of antibodies elicited by a 3rd-dose booster of an inactivated vaccine. (A) Plasma neutralizing activity evaluated by authentic SARS-CoV-2 and (B) pseudo-typed SARS-CoV-2 neutralization assays. Left: half-maximal neutralizing titer (NT_50_) values for plasma from COVID-19 convalescents, 2-dose, 3-dose CoronaVac vaccine recipients (at week 4 after the last dose of vaccination) and negative controls (pre-COVID-19 historical control) against live SARS-CoV-2 WT, B.1.351, P.1 and B.1.617.2, and VSV-based SARS-CoV-2 pseudoviruses bearing WT or B.1.1.7 or P.1 S protein. Black bars and indicated values represent geometric mean NT_50_ values. Statistical significance was determined using the two-tailed Wilcoxon matched-pairs test. Experiments were repeated in triplicate. Dotted lines indicate the limit of detection. Right: fold decrease in neutralization for each variant relative to WT for each cohort of plasma samples (calculated from the left datasets) is shown. (C) Longitudinal neutralizing titers of plasma from 3-dose vaccinees at days 0, 7, 14, 28, 90, and 180 post the 3rd-dose vaccination. The geometric mean NT_50_ values are labeled. (D) Kinetics of the 3rd-dose booster elicited recall response as indicated during monitoring of NAb titers at different time points. The green and blue curves show the changes in kinetics of NAb titers for pre-3rd-dose and post-3rd-dose vaccination, respectively. (E) Pie charts represent the distribution of antibody sequences from the four 3-dose vaccinees. The number in the inner circle is the number of sequences analyzed here. Pie-slice size is proportional to the number of clonally related sequences. The black outline indicates the frequency of clonally expanded sequences detected individually. Colored slices reveal clones that share the same *IGHV* and *IGLV* genes. (F) Graph shows relative clonality among seven individuals who received 2-dose or 3-dose of inactivated vaccines. Relative clonality for COVID-19 convalescents assayed at 1.3, 6.2, and 12 months after infection, as well as 2-dose mRNA vaccine recipients (Wang et al., 2021f, 2021g), previously described by Michel’s group, was compared. Black horizontal bars indicate mean values. Statistical significance was determined using two-tailed *t*-test. (G) Number of somatic nucleotide mutations in the *IGHV* (left) and *IGLV* (right) in antibodies from vaccinees, including 2-dose or 3-dose of inactivated vaccines and 2-dose of mRNA vaccines and COVID-19 convalescents assayed at 1.3, 6.2 and 12 months after infection (Wang et al., 2021f, 2021g). (H) Normalized ELISA binding (EC_50_) by antibodies isolated from the 3-dose inactivated and 2-dose mRNA vaccinees (ref) as well as COVID-19 convalescents to SARS-CoV-2 S trimer (left) and normalized pseudovirus neutralization activity (IC_50_) (right) against SARS-CoV-2 assayed at 1.3, 6.2, and 12 months after infection (ref). Among these, eight antibodies reported by Michel’s group were expressed and assessed for both binding by ELISA and pseudovirus neutralization activity for normalized comparison here. Black horizontal bars indicate mean values. (I) Pseudo-typed virus neutralization by antibodies isolated from the 3-dose vaccinees to circulating SARS-CoV-2 variants. Color gradient for bottom panel indicates IC_50_ values ranging from 0 (green), through 20 (yellow) and 200 (red) to 2,000 ng/mL (purple). Gray suggests no/very limited neutralizing activity (>2,000 ng/mL).

To further characterize the expeditiousness, longevity, and immunological kinetics of recall response stimulated by the third-dose immunization, neutralizing potencies at days 0, 7, 14, 28, 90, and 180 post the third-dose vaccination were determined (Fig. 1C and 1D). Remarkably, NAb titer surged by ~8-fold (from 7 to 53) at week 1, and peaked by ~25-fold increase (up to 177) at week 2 after the 3rd-booster and slowly decreased over time (Fig. 1C). Notably, NAb titer was maintained at around 60 on 180 days post the 3rd-booster, comparable to the high level of NAb titer elicited by the 2-dose administration (Fig. 1D). Taken together, these serological results reveal a third-dose booster can elicit an expeditious, robust and long-lasting recall humoral response.

We used flow cytometry to sort the SARS-CoV-2 S-trimer-specific memory B cells from the blood of seven selected CoronaVac vaccinees (Figs. S1–2). The gated double-positive cells were single-cell sorted and immunoglobulin heavy (*IGH*; IgG isotype) and light (*IGL* or *IGK*) chain genes were amplified by nested PCR. Overall, we obtained 422 and 132 paired heavy and light chain variable regions from S-binding IgG^+^ memory B cells from four 3-dose and three 2-dose vaccinees, respectively (Figs. 1E and S2). Surprisingly, expanded clones of cells comprised 45%–61% of the overall S-binding memory B compartment in 3-dose vaccinees, which is approximately 2-fold higher than those in COVID-19 convalescents and in mRNA or 2-dose vaccinated individuals, revealing an ongoing clonal evolution (Fig. 1E and 1F). Shared antibodies with the same combination of *IGHV* and *IGLV* genes in 3-dose vaccinees comprised ~20% of all the clonal sequences. Similar to natural infection and mRNA vaccination (Wang et al., 2021g), *IGHV3-30*, *IGHV3-53,* and *IGHV1-69* remained significantly over-represented in 3-dose vaccinees (Fig. S3). Additionally, the number of nucleotide mutations in the *V* gene in 3-dose vaccinees is higher than those in both 2-dose vaccinees and naturally infected individuals assayed after 1.3 and 6.2 months, but slightly lower than those in convalescent individuals 1 year after infection (Fig. 1G), revealing ongoing somatic hypermutation of antibody genes.

To further explore the immunogenic characteristics of the antibodies obtained from memory B cells in 3-dose vaccinees, 48 clonal antibodies, designated as XGv01 to XGv50 (no expression for XGv37 and XGv48) were expressed and their antigen binding abilities verified by ELISA (Fig. S4). Biolayer interferometry affinities (BLI) measurements demonstrated that all antibodies bound to WT SARS-CoV-2 at sub-nM levels (Table S1). The normalized geometric mean ELISA half-maximal concentration (EC_50_) revealed that all antibodies (EC_50_ = 4.5 ng/mL) obtained from 3-dose vaccinees, in particular RBD-specific mAbs (EC_50_ = 3.5 ng/mL), possessed higher binding activities than RBD-mAbs from early convalescents (at 1.3 and 6.2 months after infection, EC_50_ = 5.0 and 6.8 ng/mL, respectively) and mRNA (EC_50_ = 4.4 ng/mL) vaccinated individuals, but were comparable to those from late convalescent individuals (EC_50_ = 2.6 ng/mL) assessed at 12 months after infection (Fig. 1H). These results indicate the possibility of the loss of antibodies with low binding affinities over time or an ongoing increase in affinity under repeated exposures to antigen. Among these antibodies tested, 26 were bound to RBD, 16 targeted NTD, and 6 interacted with neither RBD nor NTD, but bound S1 (S1/non-RBD-NTD) (Table S1). Pseudovirus neutralization assay revealed that all RBD-specific antibodies, 10 (~60%) of the 16 NTD-directed antibodies and 3 (~50%) of the 6 S1/non-RBD-NTD antibodies were neutralizing, presenting a relatively high ratio for NAbs (Figs. 1I, S5 and Table S2). Authentic SARS-CoV-2 neutralization assay results largely verified their neutralizing activities, albeit higher concentrations were required for some NAbs (Fig. S6). In line with binding affinity, the normalized geometric mean IC_50_ of the RBD antibodies of 3-dose vaccinees was 80 ng/mL, substantially lower than those from naturally infected individuals (ranging from 1.3 to 6.2 months, IC_50_ = 130–160 ng/mL) and mRNA vaccinated individuals (IC_50_ = 150 ng/mL), but similar to those from late convalescents (IC_50_ = 78 ng/mL) (Fig. 1H). The overall increased neutralizing potency might have resulted from the ongoing accumulation of clones expressing antibodies with tight binding.

RBD is one of the main targets of neutralization in SARS-CoV-2. RBD exists in either an “open” or “closed” configuration (Walls et al., 2020), bearing antigenic sites with distinct “neutralizing sensitivity.” To dissect the nature of the epitopes of RBD targeted by NAbs, 171 SARS-CoV-2 RBD-targeting NAbs with available structures (Lv et al., 2020; Zhou et al., 2021), including cryo-EM structures determined in this manuscript (Figs. S7–8 and Table S3), were examined. By using cluster analysis on epitope structures, the antibodies were primarily classified into six sites (Ⅰ, Ⅱ, Ⅲ, Ⅳ, Ⅴ, and Ⅵ) (Figs. 2A and S9). Additionally, we superimposed structures of RBDs from these complex structures and calculated the clash areas between any 2 NAbs (Fig. 2B). Both strategies yielded identical results. Combining the results of the characterization of binding and neutralization studies reported previously with those determined here, the key structure-activity correlates for the six classes of antibodies were analyzed (Fig. 2). Antibodies with sites Ⅰ, Ⅱ, and Ⅲ, most frequently elicited by SARS-CoV-2 early infection, target the receptor-binding motif (RBM), and potently neutralize the virus by blocking the interactions between SARS-CoV-2 and ACE2 (Fig. 2C and 2D). Class I antibodies, mostly derived from *IGHV3-53*/*IGHV3-66* with short HCDR3s (generally < 15 residues), recognize only the “open” RBD, and make major contacts with K417 and N501 (Figs. 1F, 1G, and S9–10). Approximately ~75% and 60% of class I NAbs were significantly impaired in binding and neutralizing activities against B.1.351 as well as P.1, respectively, due to the combined mutations of K417N/T and N501Y (Figs. 2D, S11 and S12). Contrarily, Class III antibodies bound to RBD either in “open” or “closed” conformation, extensively associated with E484, and partially with L452 (Figs. 2D and S10C). Disastrously, over 90% of class III antibodies showed a complete loss of activity against B.1.351 as well as P.1 largely owing to an E484K mutation (Fig. S12). Against B.1.617.2, the substantially decreased activity of ~half of the class III antibodies is presumably mediated by L452R (Fig. S12). Class II antibodies use more diverse VH-genes and target the patch lying between sites I and III (Figs. 2D and S13). As expected, the effects of mutations on the activity of class II antibodies were severe, two-thirds of these antibodies had >10-fold fall in neutralization activities against VOCs (Fig. S12). Overall, the above analysis reveals that the RBD mutations identified in several VOCs can significantly reduce and, in some cases, even abolish the binding and neutralization of classes I to III antibodies, albeit being the most potent neutralizing antibodies against WT SARS-CoV-2.

**Figure 2.**
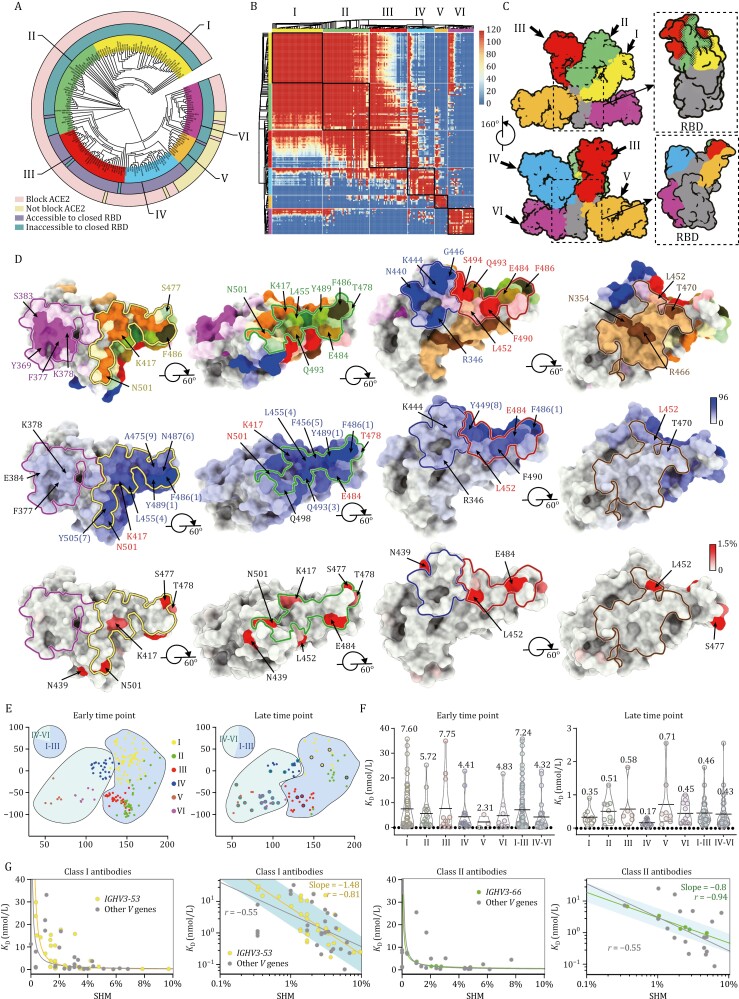
Structural, immunogenic, and evolutionary features of RBD Nabs. (A) Structure-based antigenic clustering of SARS-CoV-2 RBD NAbs. A total of 171 RBD NAbs with available structures were classified into six clusters (Ⅰ, Ⅱ, Ⅲ, Ⅳ, Ⅴ, and Ⅵ). NAbs that can block ACE2 binding or not are outlined in light pink and light yellow, respectively. NAbs that can attach to the closed RBD or not are outlined by gray blue and gray green, respectively. (B) Superimposition matrix of 171 RBD NAb structures’ output from clashed areas (Å^2^) between variable regions of any two Fab fragments showing the clustering into six antibody classes. (C) Surface representative model of six types of NAbs bound to the RBD. Fab fragments of six representative antibodies are shown in different colors and the RBD is colored in gray. Insets illustrate the antigenic patches targeted by six representative antibodies. Dashed dots indicate the overlaps between two adjacent antigenic patches. (D) Structural landscapes of the six classes of RBD NAbs (upper panel). Antigenic patches (with targeting frequency > 30%) recognized by six classes of NAbs are outlined in the assigned color scheme (same as Fig. 2C), among which residues with “hot targeting frequency” (generally over 65%, but over 85% in class I) are shown in bright colors corresponding to the patches they belong to. Residues involved in two (such as Y489, L452) or three (such as F486) neighboring antigenic patches are presented in a mixed color. Representative “hot” antigenic residues are labeled. Middle: hot map for antigenic residues on the RBD. Per residue frequency recognized by the 171 NAbs were calculated and shown. The top 9 of the hottest antigenic residues and key residues with substitutions in several VOCs are marked and labeled. Bottom: hot map for circulating variants with mutations on the RBD. Mutation frequency for each residue was calculated based on the datasets from GISAID. (E) Uniform manifold approximation and projection (UMAP) plot displaying the antibodies defined as the early time point group (left) and late time point group (right). The antibodies are colored based on their cluster assignments by the hierarchical clustering algorithm. Antibodies from I to III and IV to VI are highlighted in cyan and gray blue background, respectively. Pie charts represent the frequency distribution of antibodies belonging to I to III and IV to VI. Antibodies isolated from 3-dose vaccinees are outlined by black lines. (F) Dissociation constants (*K*_D_) of the antibodies from I to VI. Individual class antibodies are represented in colors corresponding to the classes they belong to. The color scheme is same as Fig. 2E. BLI traces are shown in Fig. S9. (G) The measured *K*_D_–SHM plots (left) and *K*_D_–SHM log-log plots (right) of antibodies from I and II are shown. *IGHV3-53* and *IGHV3-66* antibodies belonging to classes I and II are colored in yellow and green, respectively. The straight curves and lines are the least squares fits of the data to the power law with the values of the slope for *IGHV3-53* and *IGHV3-66* antibodies. The black curves and lines indicate the fitting of antibodies from I or II; the yellow and green ones suggest the fitting of *IGHV3-53* and *IGHV3-66* antibodies, respectively. The cyan lines are the 90% predicted interval.

By contrast, antibodies of the other three classes recognize evolutionarily conserved regions distinct from the RBM and some of these are often cross-reactive with other sarbecoviruses (Fedry et al., 2021). The binding of class IV antibodies, albeit attached to the apical shoulder of the RBM, is focused on a condensed patch that comprises residues 345–346, 440–441, 444–446, 448–450, which are not related to mutations observed in early VOCs (Figs. 2C and S9). Interestingly, class IV antibodies can execute their neutralizations via multiple mechanisms (Barnes et al., 2020a). Class IV antibodies, e.g., 1-57, 2-7, S309, and BD-812, hold the greatest potential for harboring markedly high tolerance to most VOCs. Site V is located beneath the RBM ridge, opposite to the site I, and adjacent to the site III. None of the class V antibodies compete with ACE2 binding (Figs. 2D and S10). Due to ~40% targeting frequency to L452, B.1.617.2, but not other VOCs, partially decreased the activities of some class V antibodies (Fig. S12). Class VI antibodies recognize a patch on one side of the RBD, distal from the RBM. Among these, some compete with ACE2 binding, while some do not, and this largely depends on the orientation/pose of the antibodies bound. In spite of less potency, antibodies targeting sites V to VI are mostly tolerant to the VOCs.

To further understand the drivers of viral evolution, we constructed immunogenic and mutational heatmaps for RBD using the 171 NAb complex structures to estimate *in vivo* NAb-targeting frequencies on the RBD and viral mutation frequencies (calculated from the datasets in the GISAID), respectively (Figs. 2D and S13). Immunogenic heatmap revealed that the epitope residues of sites I to III showed predominantly higher NAb recognition frequencies (about 53.8, 55.0, and 49.2 antibodies per residue on average for site I, II, and III, respectively) compared with those of sites IV to VI (about 19.4, 9.1, and 14.3 antibodies per residues on average for site IV, V, and VI, respectively), suggesting that class I to III antibody epitopes are “hot” immunogenic sites (Figs. 2D and S13). In line with this, residues within sites I to III exhibited dramatically higher mutation frequencies, as revealed in circulating variants that include mutations of K417, L452, S477, T478, E484, and N501 residues (Figs. 2D and S13). Surprisingly, none of the top 9 hottest immunogenic residues had a high mutation frequency. In particular, residues, such as F486, Y489, Q493, L455, F456, etc. with large side chains exhibited extremely low mutation frequencies in circulating SARS-CoV-2 strains (Figs. 2D and S14). It is worth noting that all these residues are extensively involved in the recognition of ACE2. Thus, genetic, structural, and immunogenic analysis explains why mutations at these positions would not be selected.

To investigate whether changes in the frequency of distribution of the six types of RBD antibodies are associated with evolution time, we collated and categorized human SARS-CoV-2 NAbs from available literature. For antibody clustering, we combined structural and square competition matrix analysis for 273 RBD NAbs in total (Figs. 2E and S15). In the earliest documented studies (before Dec 2020), NAbs belonging to classes I to III were predominantly identified in early COVID-19 convalescent and 2-dose vaccinated individuals (defined as early time points), accounting for up to ~80% of total antibodies. By contrast, a low ratio of NAbs from IV to VI was reported possibly due to their less potent activities at the early time point (Fig. 2E). In a series of literature (after December 2020), NAbs with enhanced neutralizing potency and breadth from IV to VI have substantially been enriched in the late convalescents or 3-dose vaccinees, almost equal in frequency to antibodies from I to III and further becoming ascendant in individuals immunized with 3 doses of inactivated vaccine (Fig. 2E). These results suggest that memory B cells display clonal turnover after about 6 months, subsequently resulting in changes in the composition of antibodies in B cell repertoire and thereby partially contributing to enhanced activities of antibodies secreted in the plasma over time. To explore the underlying mechanism, we measured the binding affinities of 167 type-classified antibodies that are also further categorized into early and late time point groups (Table S1). For the late time group, there was a 10–20 fold increase in binding affinity for individual classes, compared to those in the early time point group (Fig. 2F). In the early time point group, antibodies from IV to VI exhibited higher binding affinities to the RBD than those from I to III, in particular, antibodies from V and VI despite limited numbers (Fig. 2F). Thus, most antibodies from V and VI with low affinities and activities might be screened out in the early time point. In the late point group, sub-*nM* binding affinities for individual class antibodies with no distinct variations were observed, reflecting ongoing affinity maturation over time (Fig. S12). Our antibody clustering and *V* gene usage analysis suggests that individual class antibodies can be derived from multiple *V* genes and the shared *V* gene antibodies belong to different classes. To decipher the intrinsic trends in the relationship between binding affinity and somatic hypermutation (SHM) rate, we determined the relative affinity (*K*_D_) and calculated the SHM rate of antibodies that are encoded by the same *V* gene and belong to the same class. The measured *K*_D_–SHM plots and *K*_D_–SHM log-log plots of class I antibodies (*n* = 61), including 32 NAbs derived from *IGHV3-53*, show least squares fitting of data to a power law with a strong correlation of −0.81 for *IGHV3-53* antibodies (−0.55 for all class I antibodies) (Fig. 2G). The absolute value of its slope corresponding to a free energy change per logarithm (base e) *SHM* of cal ∙ nmol^−1^, where free energy change is 4.98*RT* + 1.48*RT* ln(*SHM*) (*R* = 2.0 cal ∙ K^−1^ ∙ nmol^−1^ and *T* = 298 K). Antibodies with adequate numbers tested from II and III exhibited similar trends by following a power law, among which *IGHV3-66* antibodies in class II yielded a compelling correlation of −0.94 despite six plots involved in the fitting (Fig. 2G). These trends indicate that as the SHM increases, the binding energy increases and *K*_D_ value decreases.

As of the initial submission of this manuscript, the B.1.617.2 variant had contributed to another surge in COVID-19 cases worldwide. Afterward, skyrocketing cases of breakthrough infections among fully vaccinated people were widely observed when other variants emerged and spread, including Omicron and its subvariants, such as BA.2, BA.5, XBB, and EG.5.1 (Cao et al., 2022b; Wang et al., 2023). Our results demonstrate that a third-dose booster of inactivated vaccine can elicit an expeditious, robust, and long-lasting recall humoral response which continues to evolve with ongoing accumulation of somatic mutations, emergence of new clones, and increasing affinities of antibodies to antigens, conferring enhanced neutralizing potency, and breadth. With the emergence of Omicron subvariants which have been evolving to largely alter viral antigenicity over time, the immune responses induced by repeated immunization of nearly all types of prototype virus-derived vaccines have been dampened. However, the immunization of variant strain-derived booster dose has shown a highly improved immune response to inhibit the viral infection and has been recommended by FDA, signifying the importance of booster dose immunization against SARS-CoV-2 (Chalkias et al., 2022). More importantly, similar to our observed antibody dynamics after the booster dose, multiple independent studies have pointed out that the strengthened immunity against the SARS-CoV-2 variants conferred by the booster dose is largely due to the sustained somatic hypermutation and spike protein-driven affinity maturation of memory B cell receptor-encoded antibodies (Muecksch et al., 2022; Zhao et al., 2022). Collectively, our findings rationalize the use of 3-dose vaccination regimens, providing fundamental insights into the clinical application of booster dose, effectively inhibiting the circulation of SARS-CoV-2 across the world.

## Supplementary Material

pwae033_suppl_Supplementary_Materials

## Footnotes

We thank Dr. Xiaojun Huang, Dr. Boling Zhu, Dr. Lihong Chen, Dr. Xujing Li, and Dr. Gang Ji for cryo-EM data collection, the Center for Biological Imaging (CBI) in the Institute of Biophysics for EM work and thank Dr. Yuanyuan Chen, Zhenwei Yang for technical help with BLI experiments. We also thank Prof. David Ho and Prof. Barton Ford Haynes for the generous gift of the sequences of two reference mAbs.

All authors have no competing interests.

This work was supported by the Ministry of Science and Technology of China (CPL-1233 and SPRG22-003), CAS (YSBR-010) and the National Science Foundation Grants (12034006,32325004 and T2394482). Xiangxi Wang was supported by National Science Fund for Distinguished Young Scholar (No. 32325004) and the NSFS Innovative Research Group (No. 81921005).

X.W., Q.W., Y.W., X-S.X., and Y.C. conceived, designed, and coordinated the experiments. Y.Z., M.X., A.Y., Y.W., Q.Z., and J-B.W. cloned and produced antibodies. K.W., Z.J., K.F., J.M., Y.J., and L.Q. expressed and purified all recombinant antigen proteins used in this manuscript. Z.J., J-J.W., Y.H., L.W., J.L., X.G., Y-J.Z., H.Z., and B-S.L. performed pseudovirus and authentic virus neutralization experiments. Z.J., K.W., L.W., P.L., W.J.F, N.W., R.C., and L.Z. performed structural study. Y.C., Y.Z., W.W., and Y.G. prepared PBMCs and flow cytometry sorting. Y.C. and A.Y. performed 10× sequencing library construction. Z.J. and K.F. performed BLI assay. Y.H. W.H. and Q.G. recruited volunteers and coordinated the collection of blood samples. All authors analyzed data; X.W. wrote the manuscript with input from all authors.

Cryo-EM density maps of the SARS-CoV-2 S trimer in complex with XGv013 or XGv043, the SARS-CoV-2 S trimer in complex with XGv004, XGv030 and XGv016; the SARS-CoV-2 S trimer in complex with XGv026 and XGv046, and the SARS-CoV-2 S trimer in complex with XGv018, XGv038 and XGv042 have been deposited at the Electron Microscopy Data Bank with accession codes EMD-38755, EMD-37680, EMD-37686, EMD-37689 and EMD-37688, respectively.
