# Supplementary material for: A third dose of inactivated vaccine augments the potency, breadth, and duration of anamnestic responses against SARS-CoV-2
Source: Protein Cell. 2024 May 27;15(12):930–7. doi: 10.1093/procel/pwae033 (PMC11637481; doi:10.1093/procel/pwae033)
Supplement: pwae033_suppl_Supplementary_Materials [file pwae033_suppl_supplementary_materials.pdf]

Supplementary Materials

A third dose of inactivated vaccine augments the potency, breadth, and duration of anamnestic responses against SARS-CoV-2

Authors: Zijing Jia[1$], Kang Wang[1$], Minxiang Xie[2$], Jiajing Wu[4$], Yaling Hu[5$], Yunjiao Zhou[10$]*, Ayijiang Yisimayi[3], Wangjun Fu[1], Lei Wang[1], Pan Liu[1], Kaiyue Fan[1], Ruihong Chen[1,6], Lin Wang[5], Jing Li[5], Yao Wang[3], Xiaoqin Ge[5], Qianqian Zhang[2], Jianbo Wu[2], Nan Wang[1], Wei Wu[2], Yidan Gao[2], Jingyun Miao[7], Yinan Jiang[7], Lili Qin[7], Ling Zhu[1], Weijin Huang[5], Yanjun Zhang[9], Huan Zhang[8], Baisheng Li[8], Qiang Gao[5], Xiaoliang Sunney Xie[3]*, Youchun Wang[4]*, Yunlong Cao[3]*, Qiao Wang[2]* and Xiangxi Wang[1,6]*

*Correspondence: X.W. (Email: xiangxi@ibp.ac.cn) or Q.W. (Email: wangqiao@fudan.edu.cn) or Y.C. (yunlongcao@pku.edu.cn) or Y.W. (Email: wangyc@nifdc.org.cn) or X.S.X. (Email: sunneyxie@biopic.pku.edu.cn) or Y.Z. (Email: centrallab5th@163.com)

[$] These authors contributed equally to this work.

This PDF file includes:

Materials and Methods

Figs. S1 to S15

Tables. S1 to S3

Materials and Methods

Facility and ethics statements

All experiments with live SARS-CoV-2 viruses were performed in the enhanced biosafety level 3 (P3+) facilities in the Sinovac Biotech Ltd approved by the National Health Commission of the People's Republic of China.

Cell lines

Vero cells (ATCC, CCL-81) and HEK293T cells (ATCC, CRL-3216) were cultured in Dulbecco's Modified Eagle's Medium (DMEM) supplemented with 10% fetal bovine serum (FBS). The cultures were maintained at 37 □ in an incubator supplied with 5% CO_2.

Viral stocks

The SARS-CoV-2 wild type strain CN01 was isolated from a patient in China during the early phase of the COVID-19 endemic. The SARS-CoV-2 variants of concern (VOC) beta (B.1.351 lineage), was isolated from a patient traveling back from South Africa; VOC gamma (P.1 lineage) was isolated from a person in Brazil; and the newly emerged VOC delta (B.1.617.1 lineage), was isolated from a traveller with PCR-confirmed infection from India in early June. All the viruses were subjected to three rounds of plaque-purifications with clones from each passage sequenced for verification as described previously (Gao et al., 2020). The final clonal of the virus was inoculated into Vero cells and grown to 95% confluence at a MOI of 0.01. When 98% of the cells developed visible cytopathic effect (CPE) after 72 hours incubation at 37 □, the cultures were harvested and centrifuged at 10,000 × g at 4 □ for 20 mins to remove the debris and preserved at -80 □ as viral stocks. Then, a small fraction of the stock was thawed at room temperature for viral titration.

Human sera

All the convalescent sera were taken from individuals recovered from COVID-19 30 – 45 days after confirmation by RT-PCR. All the vaccine sera were collected from

volunteers who received two doses or three doses of the WHO-approved inactivated SARS-COV-2 vaccine (CorovaVac, Sinovac, China).

Protein expression and purification

The plasmids encoding the full-length spike (S) protein (residues 1-1208), receptor-binding domain (RBD) (residues 319-541) and N-terminal domain (NTD) (residues 1-304) of wild-type SARS-COV-2 (GenBank: MN908947) were constructed as described previously (Lv et al., 2020). These plasmids were used as templates for the construction of S, RBD and NTD of the variants of concern - B.1.1.7 (with mutations of 69-70 del, 145 del, N501Y, A570D, D614G, P681H, T716I, S982A and D1118H), B.1.351 (with mutations of 242-244 del, L18F, D80A, D215G, K417N, E484K, N501Y, D614G and A701V), P.1 (with mutations of L18F, T20N, P26S, D138Y, R190S, K417T, E484K, N501Y, D614G, H655Y and T1027I) and B.1.617.2 (with mutations of T19R, G142D, 156del, 157del, R158G, L452R, T478K, D614G, P681R, D950N) by overlapping PCR. All the full-length S gene constructs have two proline substitutions at residues 986 and 987, a 'GSAS' substitution at the furin cleavage site and a C-terminal T4 fibritin foldon domain to facilitate the protein expression and stabilization of the trimer conformation. All the constructs described above were attached with a C-terminal twin-strep-tag II for protein purification. To obtain the S, RBD or NTD of interest, the plasmid constructed above was transiently transfected into HEK293 F cells grown in suspension at 37 □ in a rotating, humidified incubator supplied with 8% CO_2, maintained at 130 rpm. After incubation for 72 hours, the supernatant was harvested, concentrated and exchanged into the binding buffer by tangential flow filtration cassette. The protein of interest was separated by affinity chromatography using resin attached with streptavidin and further dialyzed into a buffer containing 20 mM Tris pH 8.0 and 200 mM NaCl.

Collection of Human Peripheral Blood Mononuclear Cells

Volunteer recruitment and blood draw were approved by the institutional Ethics Committee of the School of Basic Medical Sciences, Fudan University (2020-C007). Study participants, four donors receiving three doses of CoronaVac inactivated

vaccines and three donors receiving two doses, donated blood one month after vaccination. All donors ranged in age from 23-52, and the female:male ratio was 3:4. After collection of peripheral blood, human peripheral blood mononuclear cells (PBMCs) were isolated, aliquoted, and stored in liquid nitrogen.

Single Memory B Cell Sorting and Antibody Cloning

Single memory B cell sorting and antibody cloning from the sorted single B cells were performed as described previously (Zhou et al., 2021c). Briefly, stored PBMCs were thawed and incubated with CD19 MicroBeads (Miltenyi Biotec). CD19+ B lymphocytes were then incubated sequentially with human Fc block (BD Biosciences), anti-CD20-PECy7 (BD Biosciences), S-ECD-PE, and S-ECD-APC. The single memory B cells (CD20-PECy7+ S-ECD-PE+ S-ECD-APC+) were then sorted into 96-well plates using a FACSAria II (BD Biosciences), and used for antibody cloning as previously reported (Zhou et al., 2021c). Amplified PCR products of Fab regions of immunoglobulin heavy and kappa/lambda light chains were subjected to electrophoresis and Sanger sequencing. Their nucleotide sequences were analyzed by IMGT/V-QUEST and IgBlast, and the V(D)J gene segment and CDR3 sequences of each antibody were determined.

Antibody Expression

The selected antibodies were subjected to vector construction and antibody expression as reported previously (Zhou et al., 2021c). Briefly, all the cloned human monoclonal antibodies were prepared by transient transfection in mammalian HEK293F cells, which were cultured using serum-free OPM-293-CD05 medium (OPM Biosciences) at 37°C under 5% $CO2$ with shaking at 100 rpm.

Production of Fab fragment

To generate the Fab fragments, the purified Mabs XGv013, XGv043, XGv004, XGv030, XGv016, XGv026, XGv046, XGv018, XGv038 and XGv042 were processed using the Pierce FAB preparation kit (Thermo Scientific) as described previously (Lv et al., 2020). Briefly, the samples were first applied to desalination

columns to remove the salt. After centrifugation, the flow through was collected and incubated with beads attached with papain to cleave Fab fragments from the whole antibodies. Then the mixtures were transferred to protein A affinity column which specifically binds the Fc fragments of antibodies. After centrifugation, the Fab fragments were obtained and dialyzed into Phosphate Buffered Saline (PBS) (ThermoFisher, catalog #10010023).

Authentic virus neutralization assay

The plasma samples collected from both the convalescents and vaccinated volunteers were inactivated first at 56 □ for 0.5 h. The inactivated serum samples or purified mAbs were diluted by serial dilution from 1: 4 or 50,000 ng/mL with cell culture medium in two-fold steps and mixed with a virus suspension containing 100 TCID50 and incubated at 36.5 □ for 2 h. Afterwards, the mixtures were added to the 96-well plates seeded with confluent Vero cells and incubated for another 5 days at 36.5 □ in an incubator supplied with 5% CO_2. Cytopathic effect (CPE) of each well was observed and recorded under microscopes by three different individuals, and then used for the calculation of neutralizing titers by the Reed–Muench method.

Pseudovirus neutralization assay

The pseudotyped viruses were produced, aliquoted, and stored as described previously (Lv et al., 2020). The generation of pseudotyped SARS-CoV-2 variants of concern was performed similarly except using the plasmids with the corresponding S protein mutations. In vitro neutralization assay using pseudoviruses was performed as described previously (Lv et al., 2020). Briefly, the Huh-7 cells were seeded and then subjected to incubation with pseudovirus/antibody mixture for 12 hours. The antibodies were serially diluted 1: 3 in PBS for nine dilutions in total, with the beginning concentration of 10 μg/ml. Fresh DMEM medium was used to replace the mixture for further culture. After 24 or 48-hour, the Huh-7 cells were collected and luminescence was measured as described previously (Zhou et al., 2021c).

ELISA and Competition ELISA

The ELISA and competitive ELISA assays were performed as reported previously (Zhou et al., 2021c). For ELISA, 96-well plates were coated with antigen proteins (10 μg/ml) and then blocked with 2% BSA in PBS. The first antibody was serially diluted 1: 3 in PBS for eight dilutions in total, with the beginning concentration of 10 μg/ml, for incubation. Following the incubation and visualization with HRP-conjugated second antibody (Thermo Fisher Scientific), the area under the curve (AUC) was then calculated (PRISM) to evaluate the antigen-binding capacity. For competitive ELISAs, 96-well plates were coated with antigen proteins (2 μg/ml) and then blocked with 2% BSA in PBS. Incubation with first blocking antibody (15 μg/ml) was followed by directly adding biotinylated second antibodies (0.25 μg/ml). Streptavidin-HRP (BD Biosciences) was then added for detection. Samples with no first antibody were used as a negative control for normalization.

Bio-layer interferometry

Bio-layer interferometry (BLI) experiments were carried out on an Octet Red 96e machine (Fortebio). To measure the binding affinities of monoclonal antibodies with native and variant RBDs, antibodies were immobilized onto protein A biosensors (Fortebio), while serial dilutions of RBD or its variants were used as analytes. Data were recorded using software Data Acquisition 11.1 (Fortebio) and analyzed using software Data Analysis HT 11.1 (Fortebio) with a 1: 1 fitting model.

Cryo-EM sample preparation, data collection and processing

The purified S protein was mixed and incubated with the Fab fragments of XGv013 and XGv043, XGv004, XGv030 and XGv016, XGv026 and XGv046, as well as XGv018, XGv038 and XGv042 with a molar ratio of 1 : 1.5 (S protein to Fab) to obtain the S-Fab-complexes. Then, three-microliter aliquot of each complex was deposited onto the glow-discharged holey carbon-coated gold grid (C-flat, 300-mesh, 1.2/1.3, Protochips In.), blotted for 7 seconds in 100% relative humidity and plunged into the liquid ethane using Vitrobot (FEI) (3,4). Cryo-EM data sets were collected at 300 kV with a Titan Krios microscope (FEI). Movies (32 frames, each 0.2 s, total dose of 60 e^{-} $Å^{-2}$) were recorded using a K2 Summit direct detector with a defocus

range between 1.5-2.7 µm. Automated single particle data acquisition was performed by SerialEM, with a calibrated magnification of 22,500 yielding a final pixel size of 1.04 Å.

Model fitting and refinement

Coordinates for initial complexes were generated by docking individual chains from reference structures into cryo-EM density using UCSF Chimera. (S trimer, PDB number 6VXX; Fab, PDB number 7CAK). Models were then adjusted manually using Coot and automatically refined into cryo-EM maps by rigid-body and real-space refinement in Phenix.

PISA and clustering

All the published structures of SARS-CoV-2 neutralizing antibodies in complex with S trimer or its subdomain (RBD or NTD) were obtained from the Protein Data Bank and were superimposed on RBD individually. The buried surface areas (BSA) of each pairwise antibody were recorded as the distance between them. Afterwards, the clustering of BSA matrix was performed with pheatmap in R.

Epitope identification and Clustering

All the published structures of SARS-CoV-2 neutralizing antibodies in complex with S trimer or its subdomain (RBD or NTD) were obtained from the Protein Data Bank. All residues on RBDs and NTDs with distances within 4 Å from their corresponding antibodies were recorded as the epitope residues. The binding frequency of each residue was calculated for the generation of epitope heatmap. The binary binding situation of each residue (aa 319-541 for RBD and aa 2-310 for NTD) were used to calculate the distance between pairwise sequences with an in-house python script. The clustering of sequence distance matrix was performed by fetch in PHYLIP.

Prediction of NAbs' class from ELISA assay.

K-means algorithm was used to classify NAbs. Briefly, we first calculated the ratio of NAbs that didn't clash with each other for all the 6 classes. The results were stored in

the matrix *S*. Then, the data of 12 NAbs belonging to 6 known classes was utilized to generate initial seeds of the following K-means clustering. This process was achieved by MATLAB script *four_template.m.* In consideration of no such significant differences for templates data for Classes I to III, antibodies from these three classes yielded from the first round of clustering needs to be further analyzed. After that, Sum of Squares for Error (SSE) between a certain NAb and the template in the same class was calculated. NAbs corresponding to large SSE (>0.5) had a low confidence level of right clustering validity and would be reclassified. The consistency of matrix *S* and data of ELISA assay were the basis for the second round of clustering. We found the best class for the reclassified NAbs by the MATLAB script *class_abnormal.m.*

Calculation of positional mutation frequency

The frequencies of positional mutations were calculated based on the SARS-CoV-2 spike sequences deposited in GISAID by April 6th, 2021 (Elbe and Buckland-Merrett, 2017). All the sequences were aligned pairwise with the wide-type strain, the mutation frequencies for each position were calculated as the proportion of total mutations in all deposited sequences. The low quality 'X' residues were not counted as mutations.

High-throughput single-cell mRNA, VDJ, and feature barcode sequencing

As previously described, SARS-CoV-2 antigen-specific B cells from 2-dose and 3-dose CoronaVac vaccinees' PBMC were isolated through MACS and FACS, and subjected to microfluidic-based single-cell sequencing (10x Genomics, Libra-seq) to obtain 5' mRNA, VDJ, and feature barcode information. Briefly, B cells were first enriched from frozen PBMCs by negative selection using the EasySep™ Human B Cell Enrichment Kit (STEMCELL, 17954). Purified B cells were stained with antibody cocktails containing oligo-barcoded antigens (RBD and NTD, PE/APC dual labeled for each antigen) on ice for 30 minutes. Stained B cells were collected on an Astrios EQ (BeckMan Coulter) for single CD14–, CD16–, 7-AAD–, CD19+, antigen+ cells. Collected cells were subjected to 10× Chromium System (10× Genomics) according to the manufacturer's instructions for Libra-seq library construction.

Sequencing was performed on the Illumina xten platform for 2x150bp dual index sequencing.

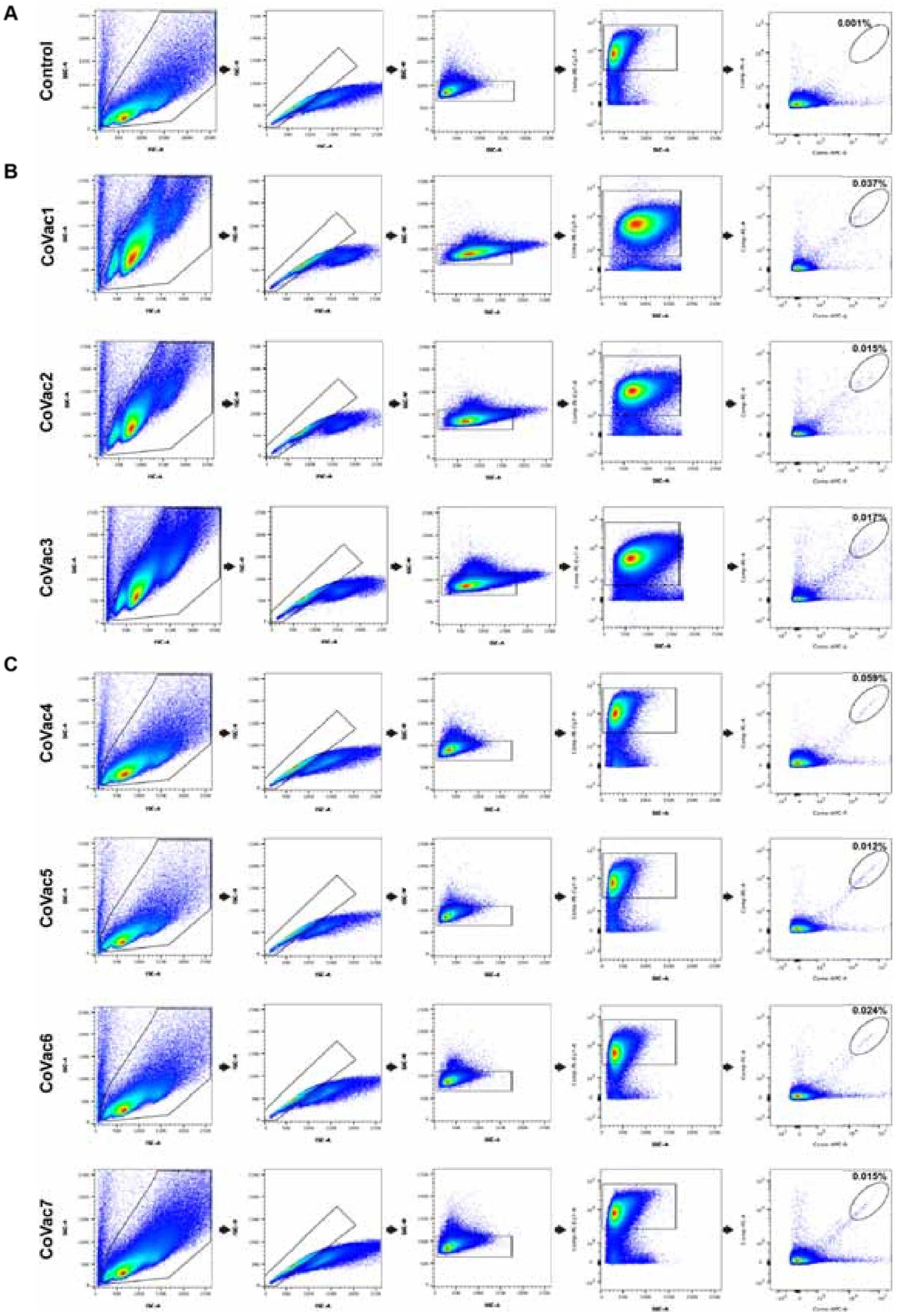

Fig S1 Gating strategy used for cell sorting and representative data for flow cytometry.

(A-C) Flow cytometry showing the percentage of S-ECD-double positive B cells from an uninfected unvaccinated control donor (A), three 2-dose vaccinees (B), and four 3-dose vaccinees. Each panel from left to right corresponds to a sequential gating strategy, from live cells (X axis, FSC-A; Y axis, SSA-A in panel 1) and singlets (X axis, FSC-A; Y axis, FSC-H in panel 2; and X axis, SSC-A; Y axis, SSA-W in panel 3) to $CD20^+$ B cells (X axis, SSC-A; Y axis, CD20-PE-Cy7 in panel 4) and S-ECD-double positive B cells (X axis, S-ECD-APC; Y axis, S-ECD-PE in panel 5). Sorted cells were $S\text{-}ECD\text{-}APC^+$ and $S\text{-}ECD\text{-}PE^+$, with their frequencies labeled in the top right corner in panel 5. FSC, forward scatter; SSC, side scatter (-A, area; -H, height; -W, width).

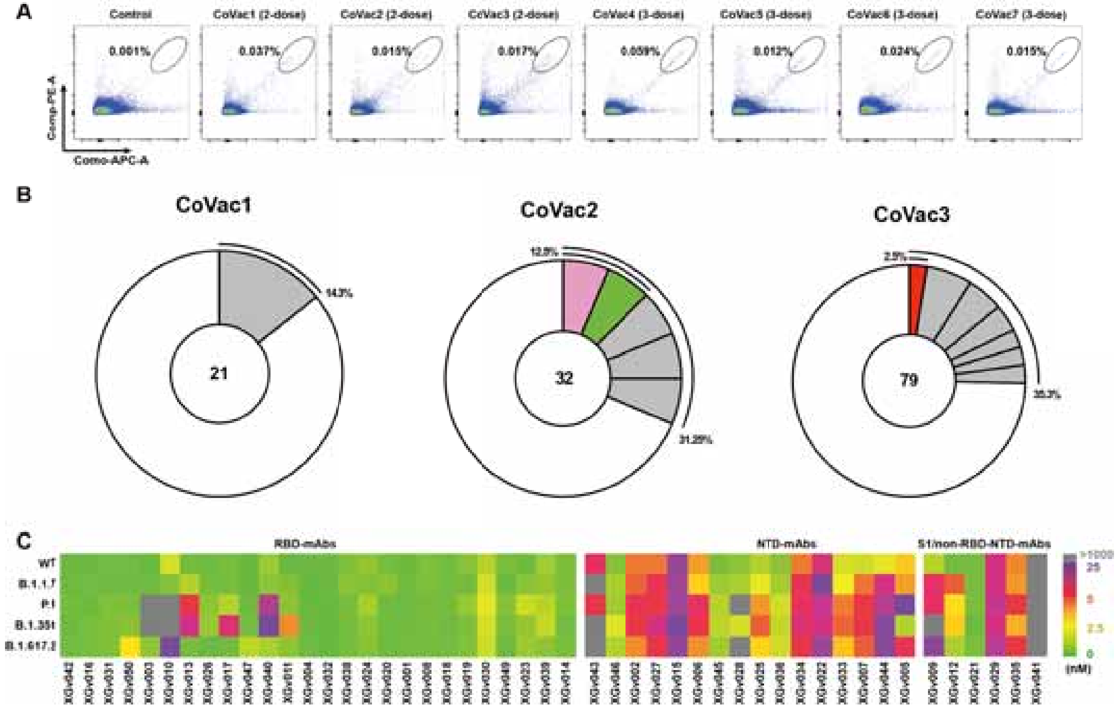

Fig. S2 Memory B cell antibodies elicited by inactivated vaccines.

(A) Representative flow cytometry plots showing dual allophycocyanin (APC)-S- and phycoerythrin (PE)-S-binding B cells for vaccinees and control donor. (B) Antibody pie charts for the three 2-dose vaccinees. There are 21, 32 and 79 sequenced antibodies with naturally paired Ig heavy and light chains, respectively for the three 2-dose vaccinees. Antibodies with the same IGHV/IGLV genes and closely related CDR3 sequences were grouped together and represented as a slice (grey). Antibody singlets are in one big slice (white). Colored slices reveal expanded antibody clones sharing the same *IGHV* and *IGLV* genes with the four 3-dose vaccinees (see Fig. 1). (C) BLI binding affinities by antibodies isolated from the 3-dose vaccinees to circulating SARS-CoV-2 variants. Color gradient indicates K_D values ranging from 0 (green), through 2.5 (yellow) and 5 (red) to 25 nM (purple). Gray suggests no/very limited binding activity (>1000 nM).

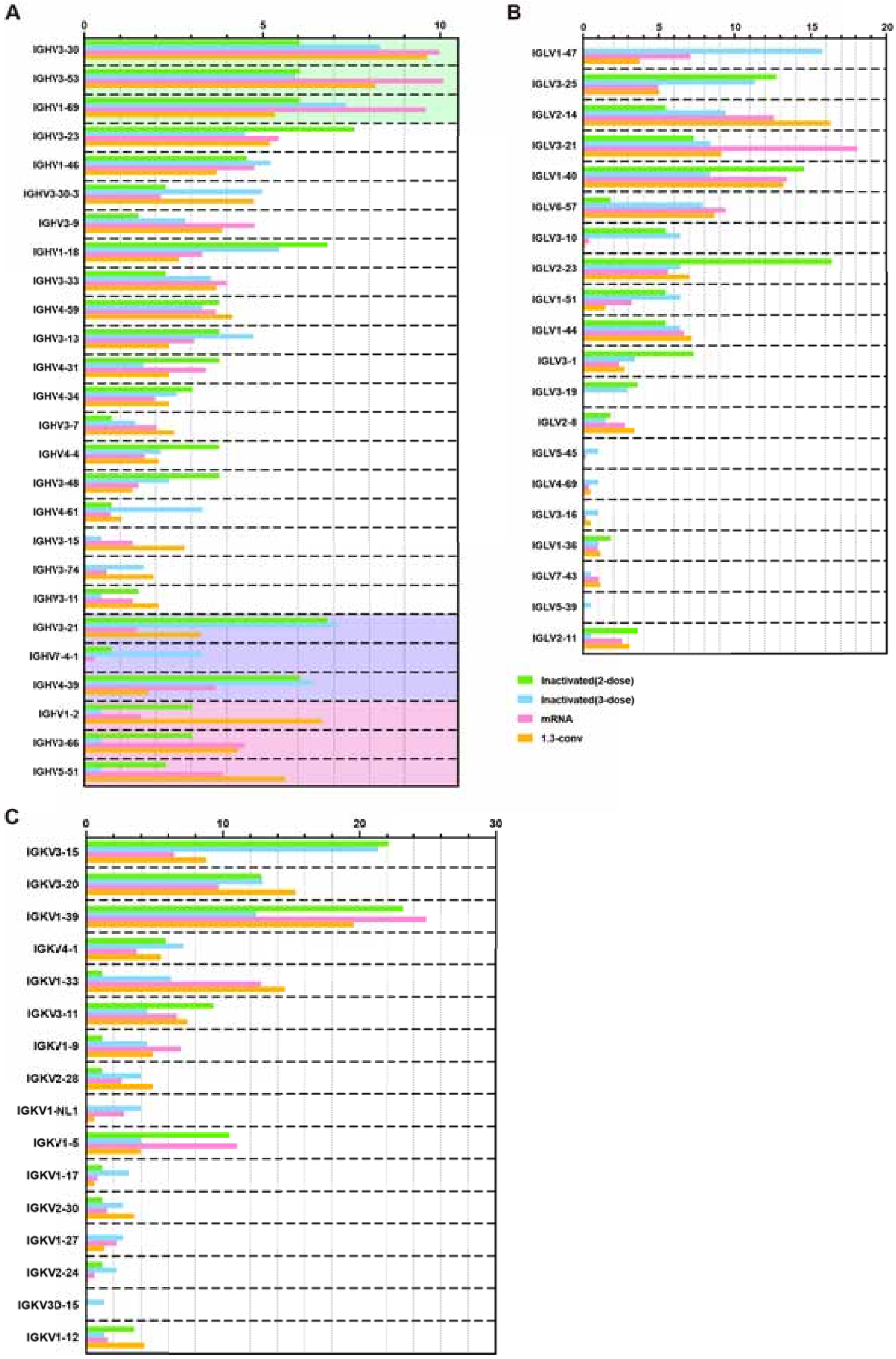

A
0
5
10
IGHV3-30
IGHV3-53
IGHV1-69
IGHV3-23
IGHV1-46
IGHV3-30-3
IGHV3-9
IGHV1-18
IGHV3-33
IGHV4-59
IGHV3-13
IGHV4-31
IGHV4-34
IGHV3-7
IGHV4-4
IGHV3-48
IGHV4-61
IGHV3-15
IGHV3-74
IGHV3-11
IGHV3-21
IGHV7-4-1
IGHV4-39
IGHV1-2
IGHV3-66
IGHV5-51
B
0
5
10
15
20
IGLV1-47
IGLV3-25
IGLV2-14
IGLV3-21
IGLV1-40
IGLV6-57
IGLV3-10
IGLV2-23
IGLV1-51
IGLV1-44
IGLV3-1
IGLV3-19
IGLV2-8
IGLV5-45
IGLV4-69
IGLV3-16
IGLV1-36
IGLV7-43
IGLV5-39
IGLV2-11
Inactivated(2-dose)
Inactivated(3-dose)
mRNA
1.3-conv
C
0
10
20
30
IGKV3-15
IGKV3-20
IGKV1-39
IGKV4-1
IGKV1-33
IGKV3-11
IGKV1-9
IGKV2-28
IGKV1-NL1
IGKV1-5
IGKV1-17
IGKV2-30
IGKV1-27
IGKV2-24
IGKV3D-15
IGKV1-12

Fig. S3 Frequency distributions of human *V* genes.

(A-C) Graphs show relative percentage (X axis) of human *IGHV* (A), *IGLV* (B) and *IGKV* (C) genes in data from convalescent (1.3 month) individuals (orange), 2-doses inactivated vaccinees (green), 3-dose inactivated vaccinees (blue), and mRNA vaccinees (pink). The top 3 popular *IGVH* genes for 3-dose inactivated, 2-dose inactivated, and mRNA vaccinees are highlighted with purple, green, and pink box, respectively.

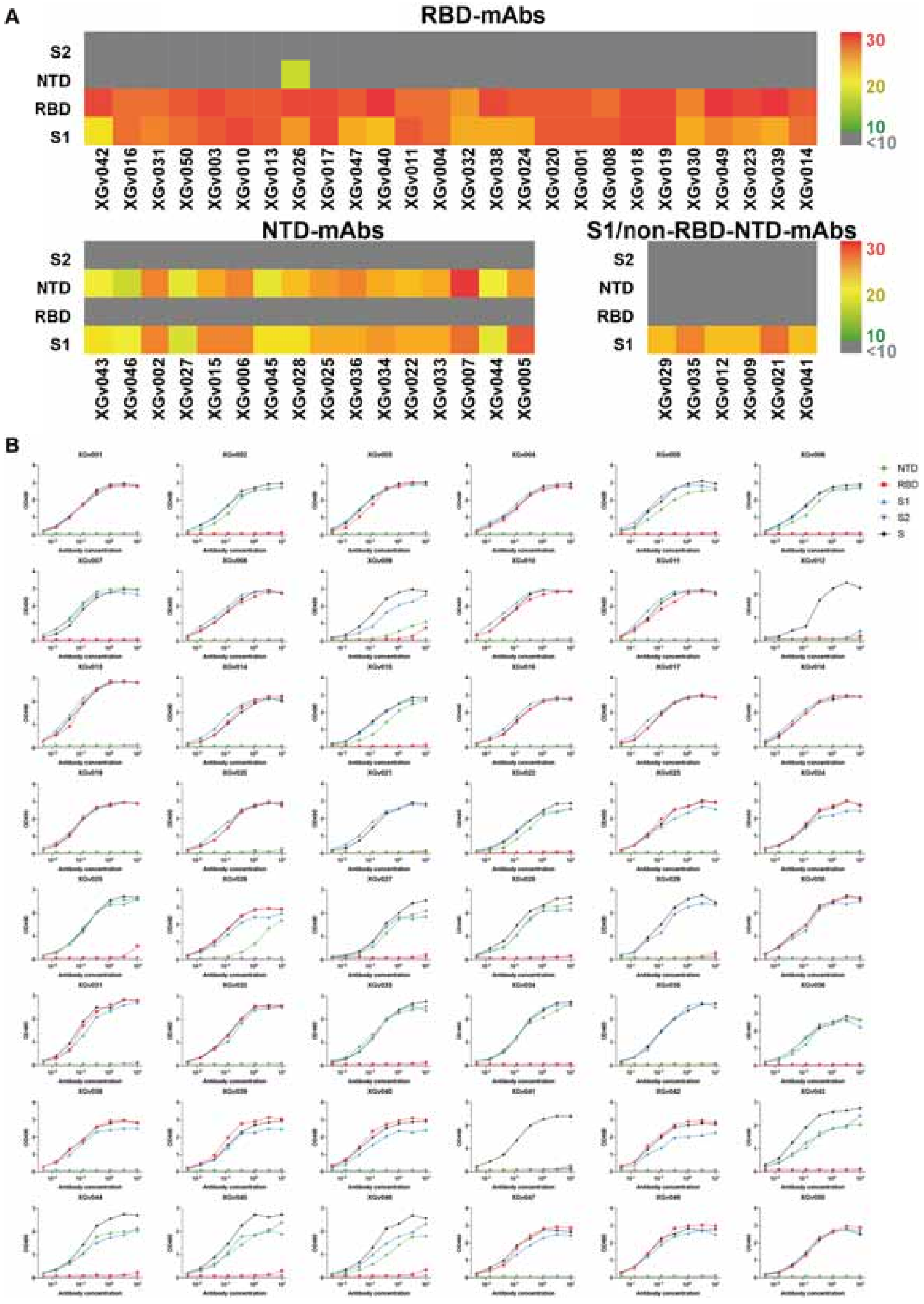
A
RBD-mAbs
S2
NTD
RBD
S1
30
20
10
<10
XGv042
XGv016
XGv031
XGv050
XGv003
XGv010
XGv013
XGv026
XGv017
XGv047
XGv040
XGv011
XGv004
XGv032
XGv038
XGv024
XGv020
XGv001
XGv008
XGv018
XGv019
XGv030
XGv049
XGv023
XGv039
XGv014
NTD-mAbs
XGv043
XGv046
XGv002
XGv027
XGv015
XGv006
XGv045
XGv028
XGv025
XGv036
XGv034
XGv022
XGv033
XGv007
XGv044
XGv005
S1/non-RBD-NTD-mAbs
XGv029
XGv035
XGv012
XGv009
XGv021
XGv041
B
NTD
RBD
S1
S2
S

C

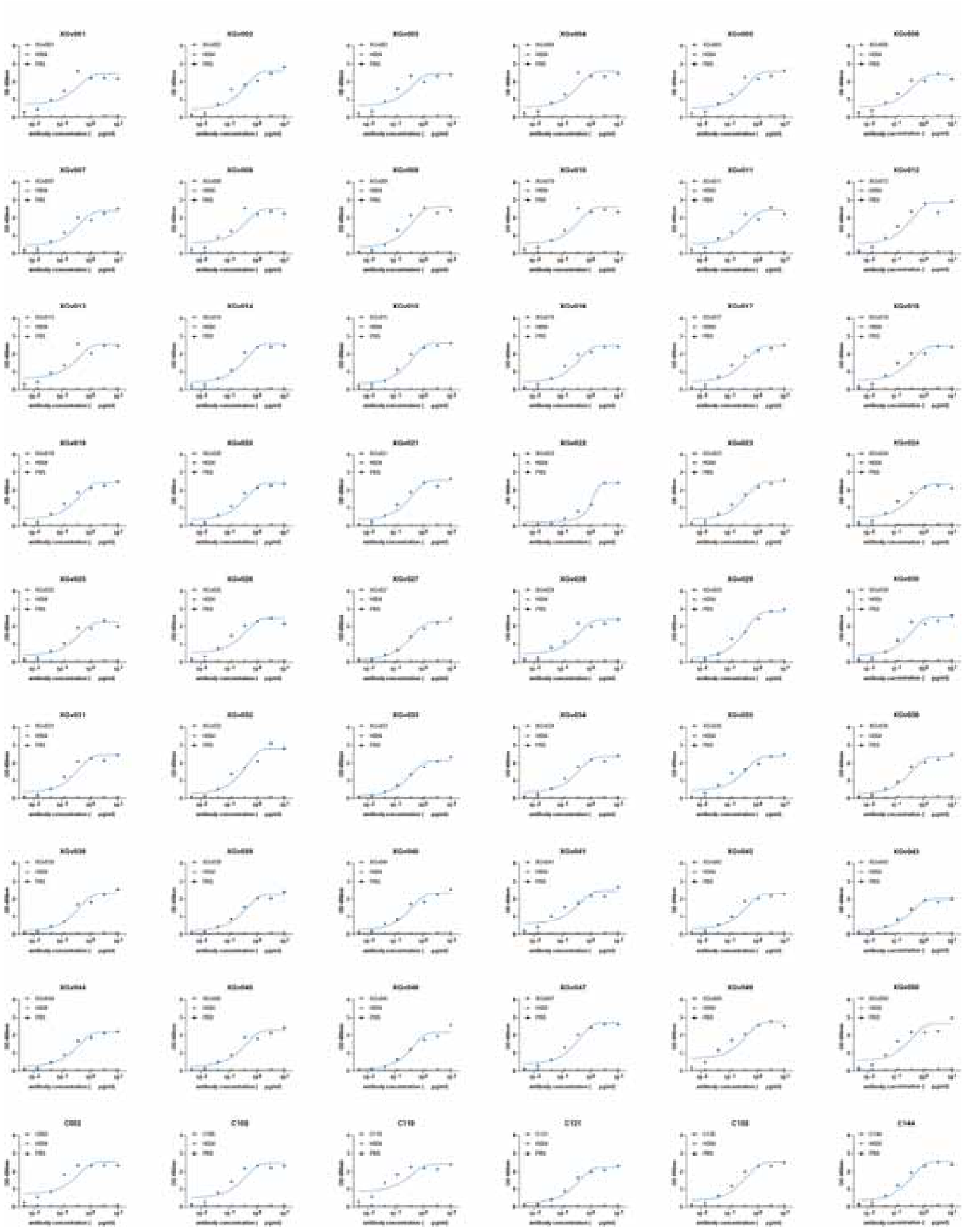

Fig. S4 Antibody binding domain scanning and EC_{50} calculation by ELISA.

(A) Heatmap representation of AUC values of the 48 XGv mAbs by ELISA against the subdomain S2, NTD, RBD or S1 of SARS-CoV-2 S, respectively was shown with

a color bar indicated on the right.

(B) Curves corresponding to each value in the heatmap were generated based on all the ELISA values at different concentrations.

(C) EC_{50} values of the 48 XGv mAbs and reference mAbs of C002, C105, C119, C121, C135 and C144 were calculated from the curves fitted from ELISA results; then, each EC_{50} of the XGv mAbs was normalized against the EC_{50} values of the reference mAbs obtained previously to compare with those of antibodies reported from Michel's group.

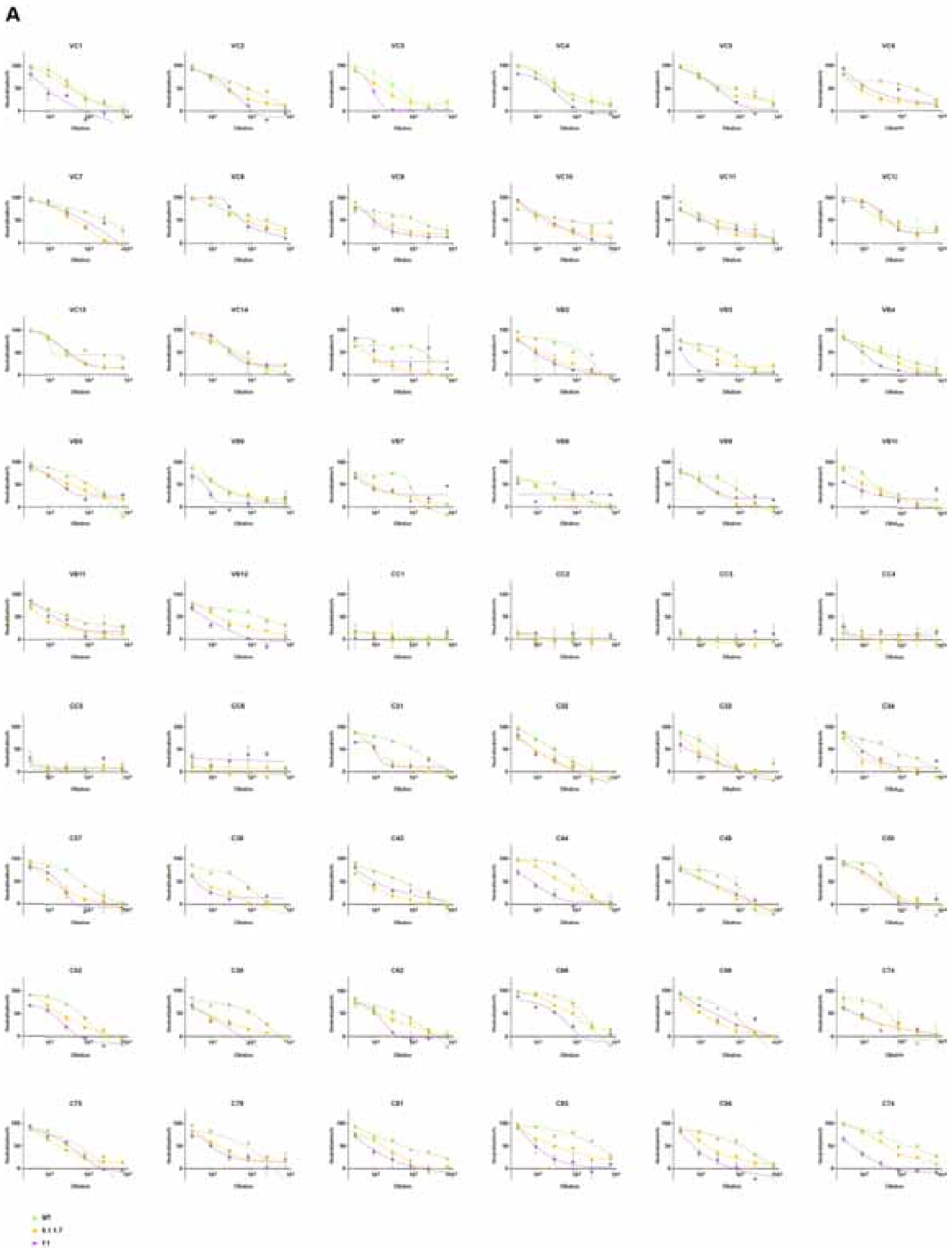

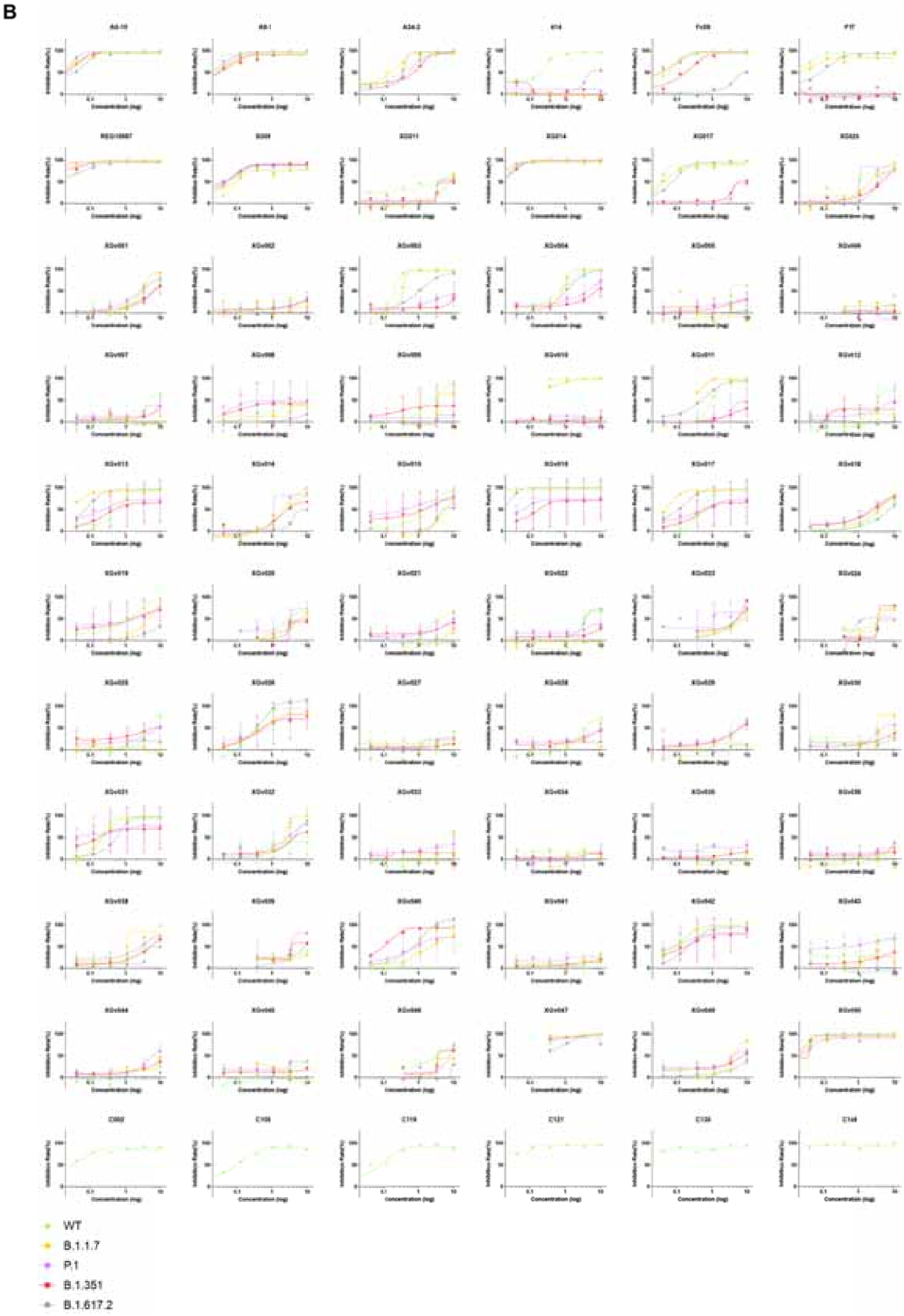

Fig. S5 Neutralization profiles of plasma and NAbs against both the WT and variant pseudoviruses.

(A) Neutralization assays of WT, B.1.1.7, B.1.351, P.1 and B.1.617.2 pseudoviruses using plasma from convalescents (C1-C22), 2-dose (VB1-VB12) and 3-dose (VC1-VC14) CoronaVac vaccinees, as well as healthy individuals (CC1-CC6).

(B) Neutralization curves of the XGv mAbs and other representative mAbs analyzed in Fig. S20 against pseudotyped viruses of both WT and VOCs. Values of each mAb are listed in Table S3.

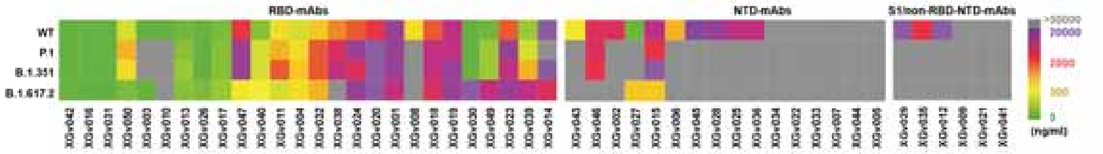

Fig. S6 Neutralization of monoclonal antibodies against authentic virus.

Neutralization activity of the 48 mAbs against pseudoviruses bearing the S protein of WT (green), B.1.351 (red), P.1 (purple), and B.1.617.2 (grey).

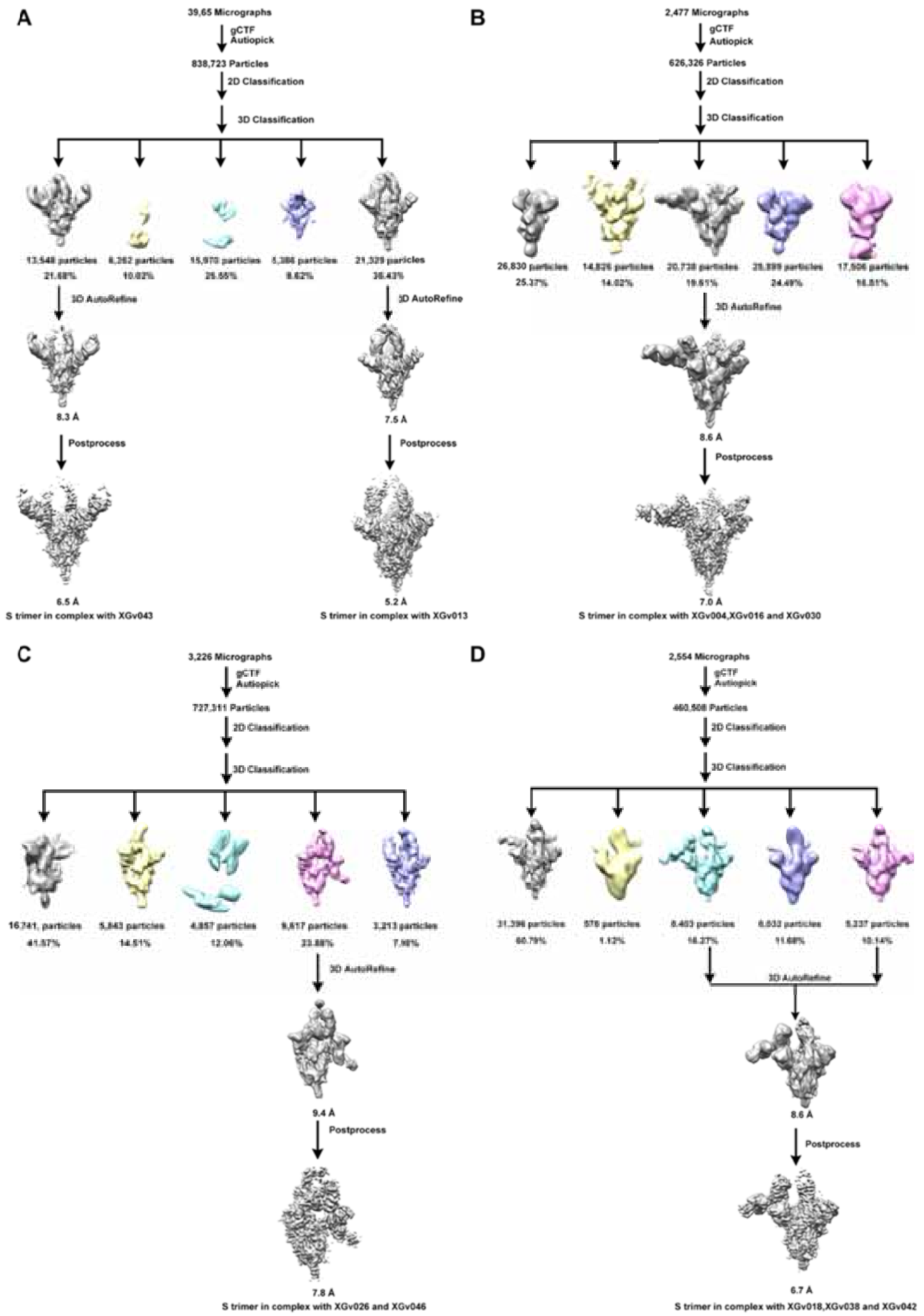

Fig. S7 Flowchart for Cryo-EM data processing.

Flowchart for SARS-CoV-2 S-XGv013-XGv043 (**A**) , S-XGv004-XGv016-XGv030 (**B**), S-XGv026-XGv046 (**C**) and S-XGv018-XGv038-XGv042 (**D**) Cryo-EM data processing are shown.

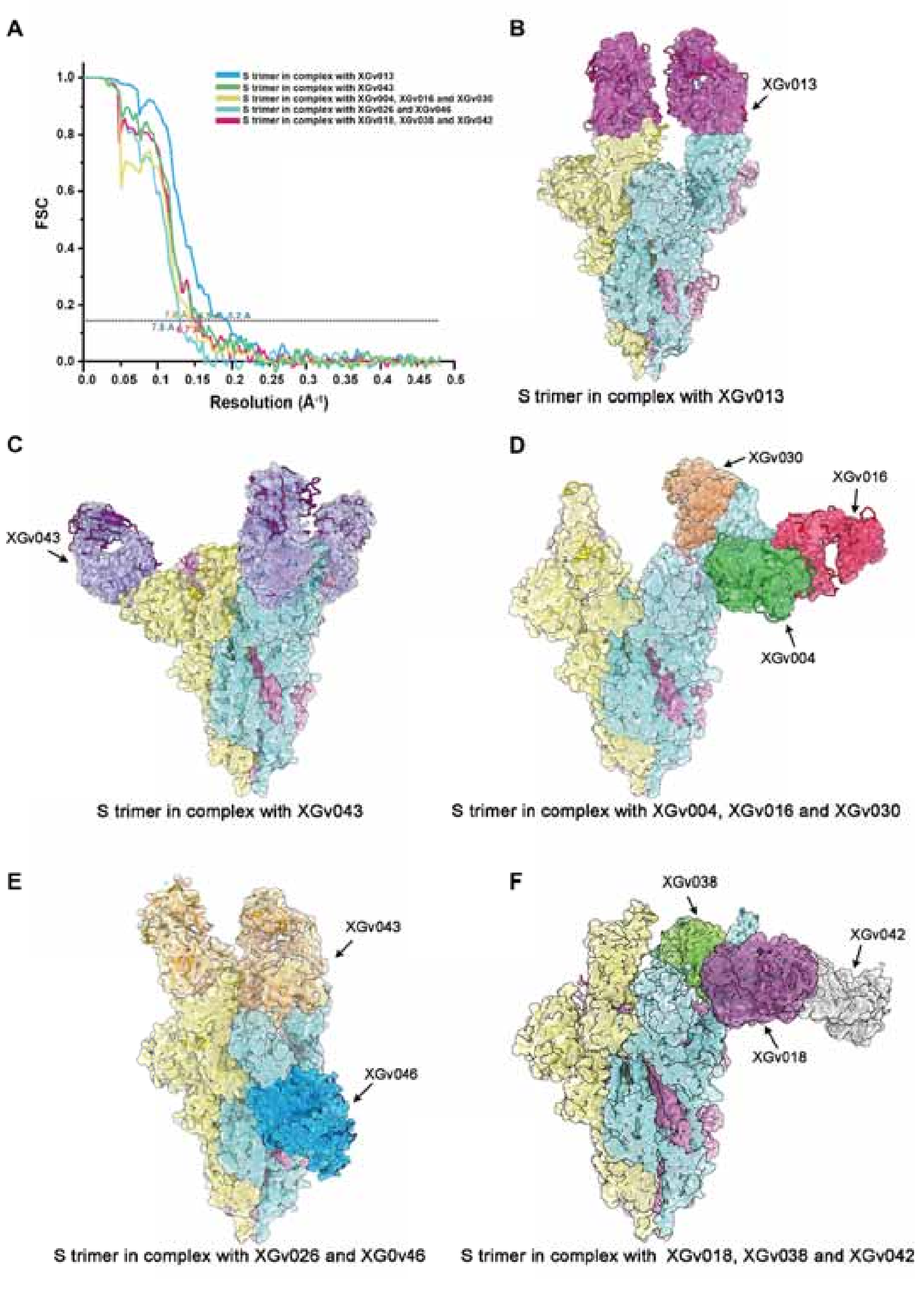

Fig. S8 Evaluation of resolution and density maps with resulting atomic models of SARS-CoV-2 S trimer in complex with Fabs.

(**A**)The gold-standard FSC curves of the final maps. (**B-F**) Cryo-EM maps of SARS-CoV-2 trimer in complex with Fabs described above. Each monomer of S trimer is colored by cyan, pink and yellow, respectively.

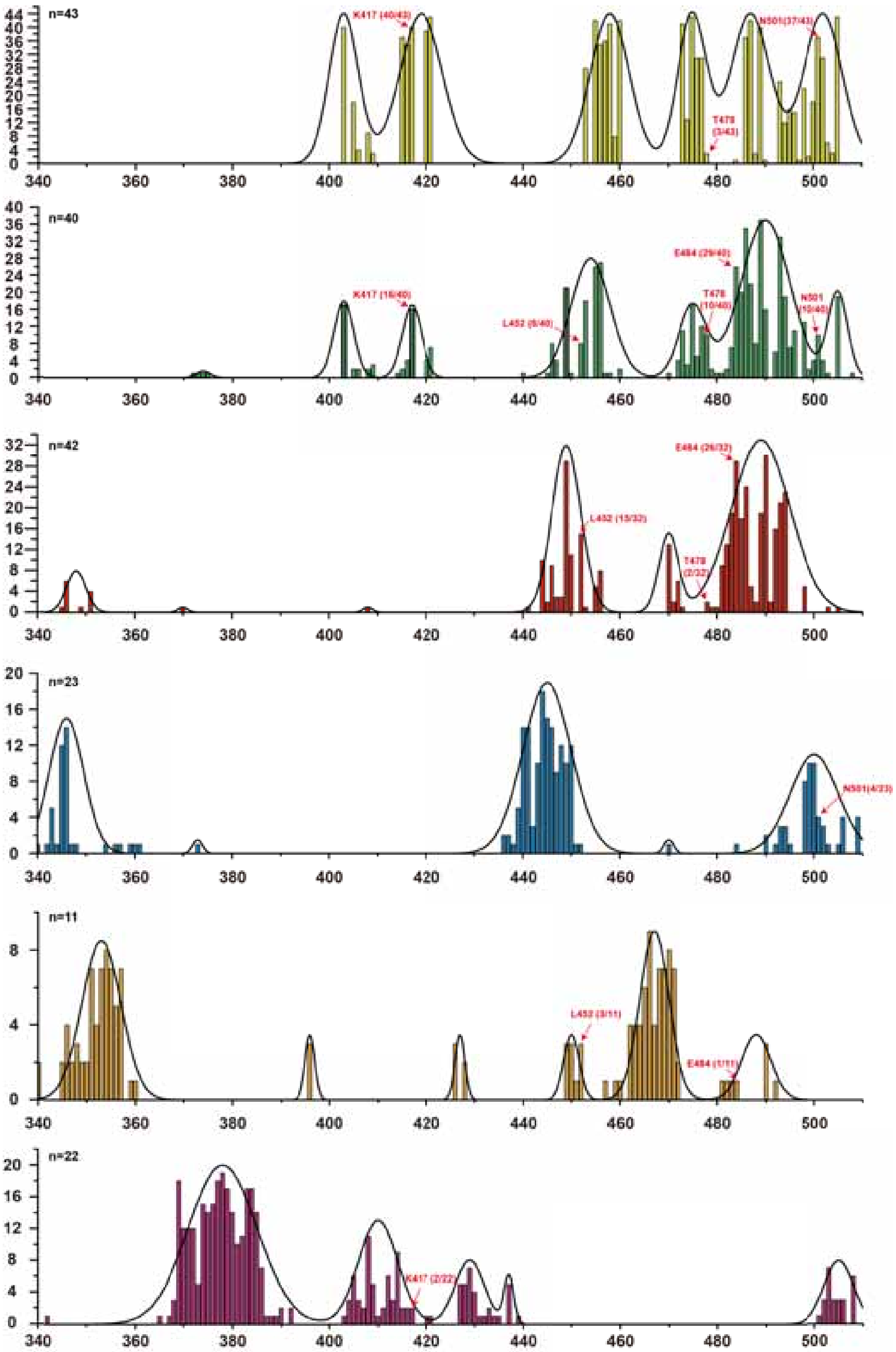

Fig. S9 Distribution of the antigenic sites for the 6 antibody classes.

Frequency of each epitope of six classes of antibodies is counted and displayed by histogram. Distribution for six antibody classes is shown by fitted curves. Color scheme is the same as in Fig. 2A.

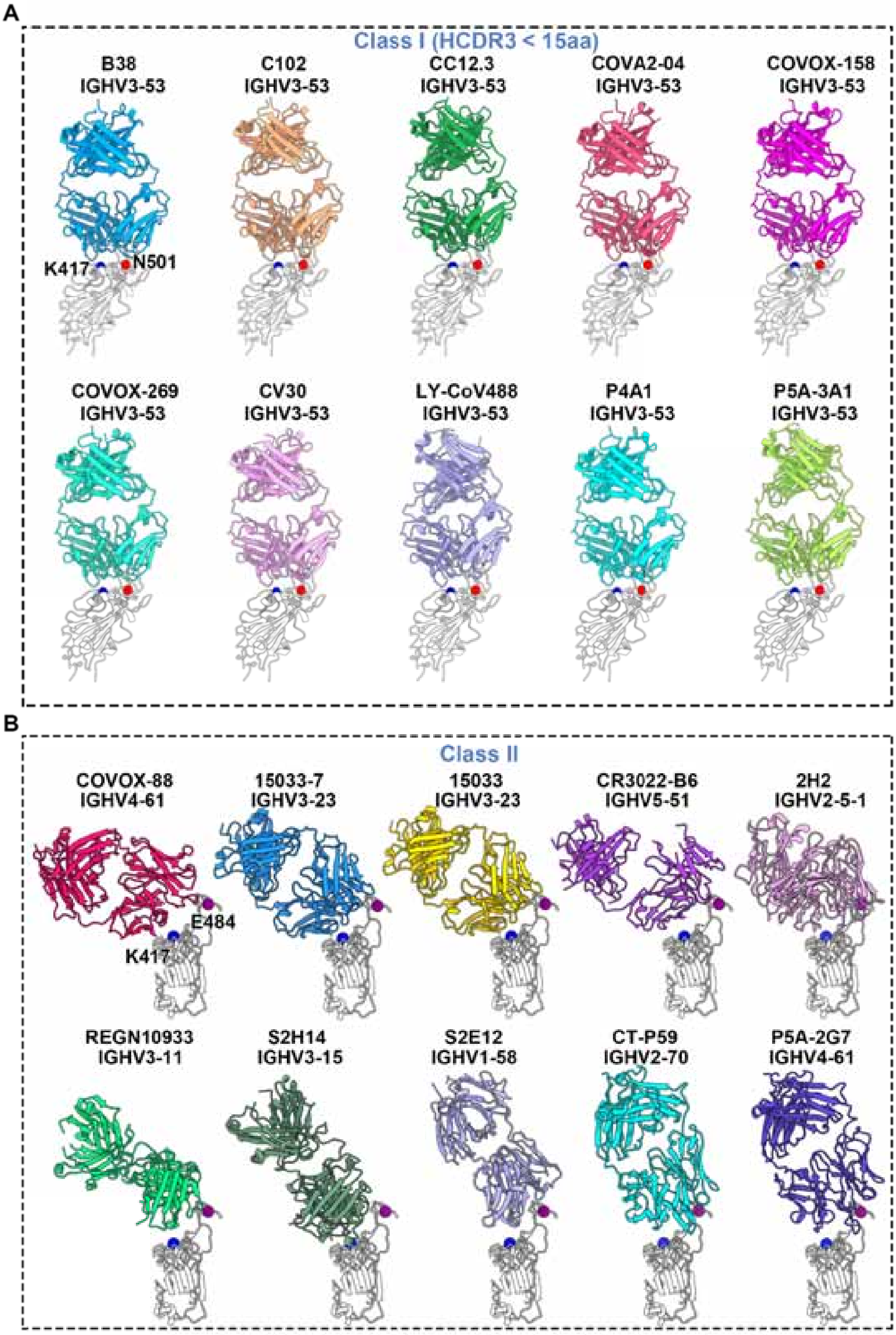

A
Class I (HCDR3 < 15aa)
B38
IGHV3-53
C102
IGHV3-53
CC12.3
IGHV3-53
COVA2-04
IGHV3-53
COVOX-158
IGHV3-53
K417
N501
COVOX-269
IGHV3-53
CV30
IGHV3-53
LY-CoV488
IGHV3-53
P4A1
IGHV3-53
P5A-3A1
IGHV3-53
B
Class II
COVOX-88
IGHV4-61
15033-7
IGHV3-23
15033
IGHV3-23
CR3022-B6
IGHV5-51
2H2
IGHV2-5-1
E484
K417
REGN10933
IGHV3-11
S2H14
IGHV3-15
S2E12
IGHV1-58
CT-P59
IGHV2-70
P5A-2G7
IGHV4-61

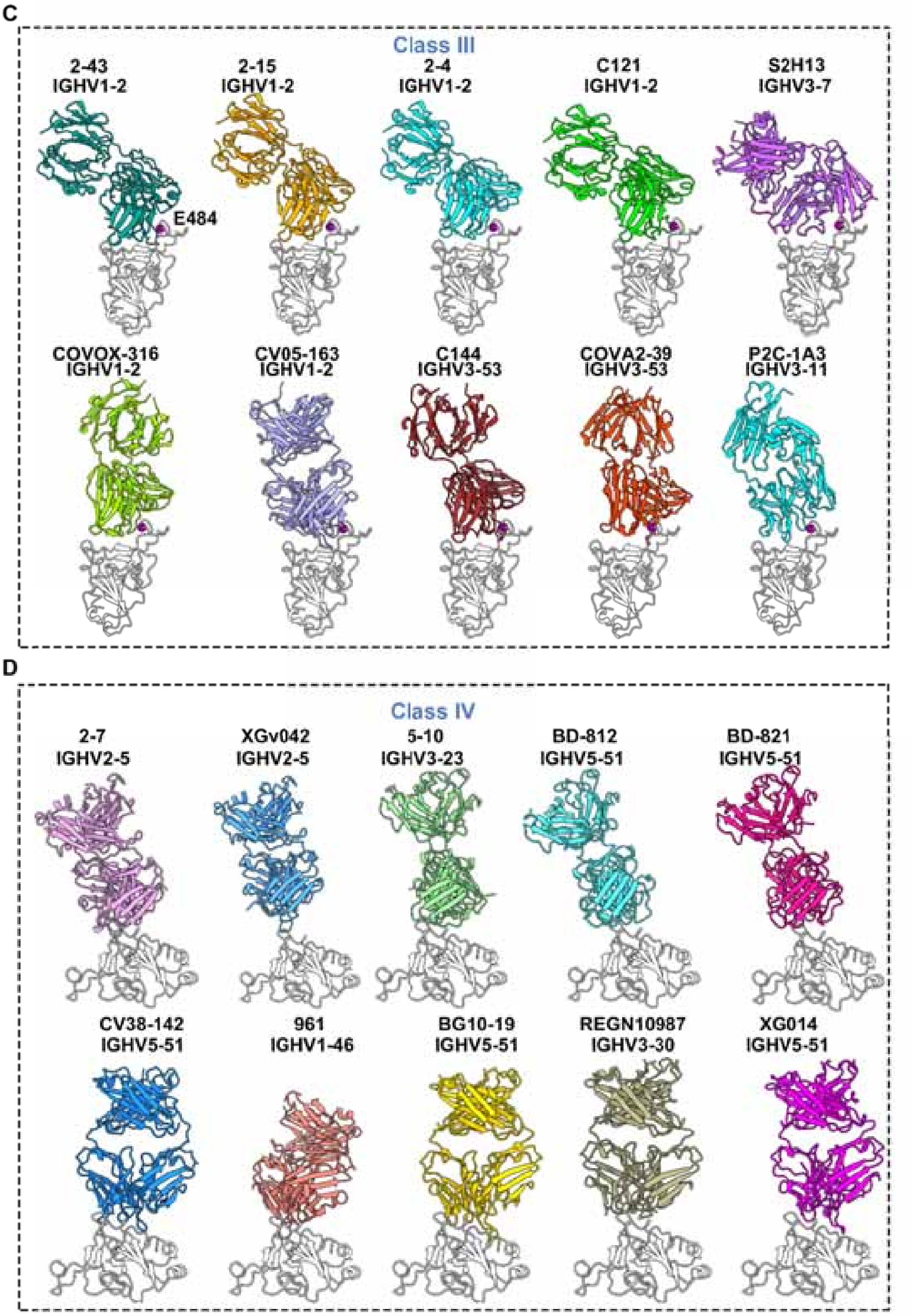
C
Class III
2-43
IGHV1-2
2-15
IGHV1-2
2-4
IGHV1-2
C121
IGHV1-2
S2H13
IGHV3-7
E484
COVOX-316
IGHV1-2
CV05-163
IGHV1-2
C144
IGHV3-53
COVA2-39
IGHV3-53
P2C-1A3
IGHV3-11
D
Class IV
2-7
IGHV2-5
XGv042
IGHV2-5
5-10
IGHV3-23
BD-812
IGHV5-51
BD-821
IGHV5-51
CV38-142
IGHV5-51
961
IGHV1-46
BG10-19
IGHV5-51
REGN10987
IGHV3-30
XG014
IGHV5-51

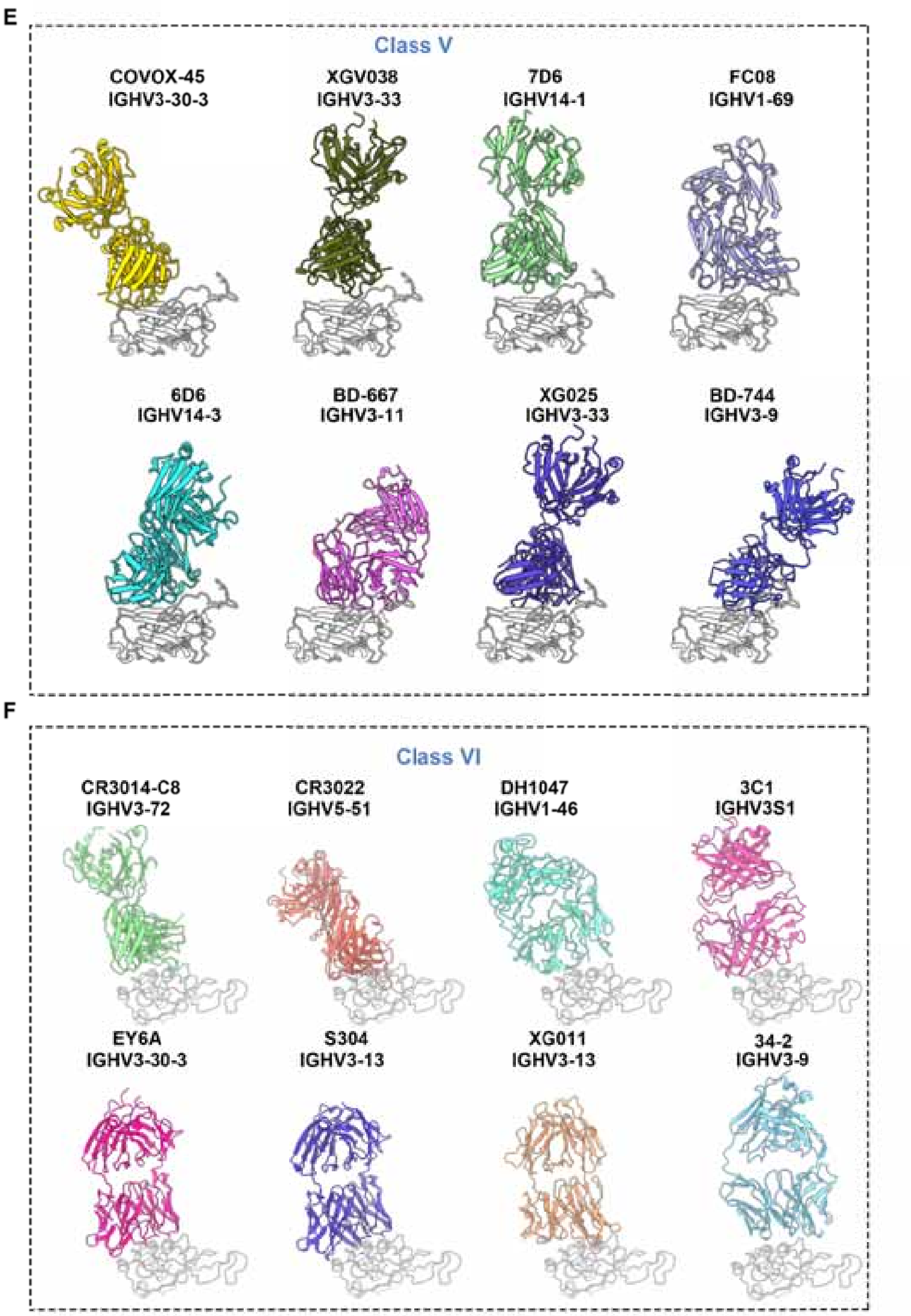

Fig. S10 Structures of 6 classes of antibodies in complex with SARS-CoV-2 RBD.
Structures of SARS-CoV-2 RBD in complex with antibodies of six classes are shown (**A-F**) as cartoon. RBD is colored by white. Residues K417, E484 and N501 are shown as sphere and colored by blue, purple and red, respectively.

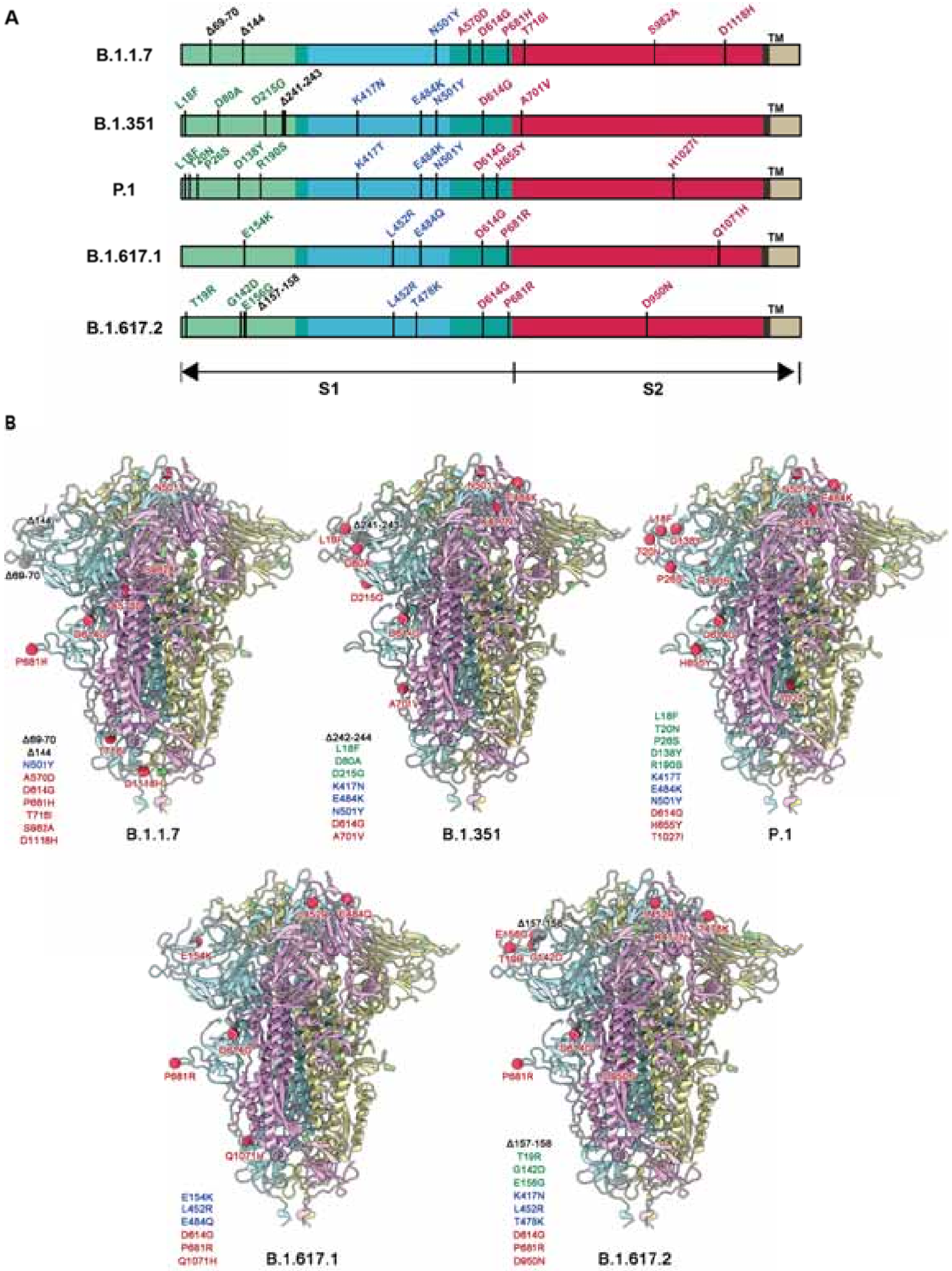

Fig. S11 Mutation residues of SARS-CoV-2 variants.

(**A**) Schematic diagram showing the mutation and deletion sites of SARS-CoV-2 variants (B.1.1.7, B.1.351, P.1, B.1.617.1 and B.1.617.2). (**B**) S trimers of variants are shown as cartoon and mutations and deletions are shown as spheres. Mutation sites are colored by red and deletion sites are colored by grey on one monomer (cyan), and both are colored by green on the other two monomers (pink and yellow).

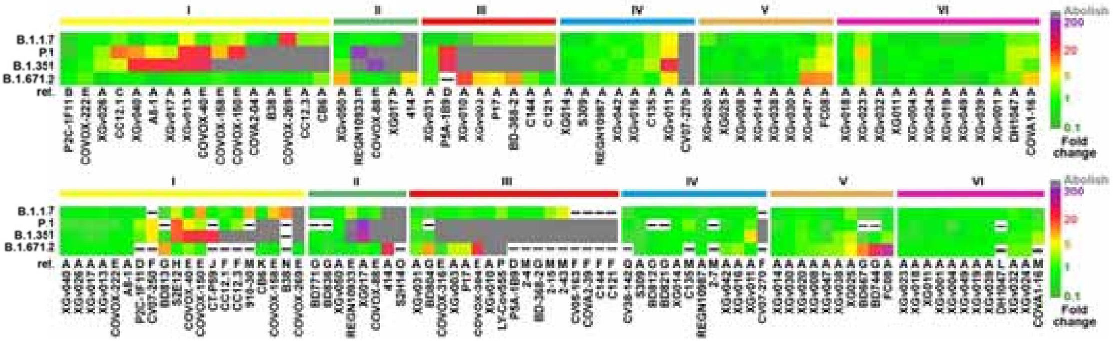

Fig. S12 Immunogenic characteristics of six classes of RBD-targeting NAbs.

Hot maps show relative fold changes in K_D values (up) and IC_{50} values (down) against several VOCs for the six classes of NAbs, including previously reported and newly isolated antibodies described in this manuscript. Color gradients for upper and bottom panels indicate relative fold changes and are shown at right side. "-": no related datasets in the original studies and related references are listed. Ref "A" indicates that the datasets were produced in this manuscript. Other letters in Ref correspond to different reference numbers shown as below. B – 91 and this manuscipt, C – 99 and this manuscript, D – 97, E – 30, 81, 103 and 104, F – 99, G – 98, H – 100 and 108, J – 101, K – 94 and 102, L – 105 and 106, M – 94, N – 105, O – 107, P – 82, Q – 66, respectively.

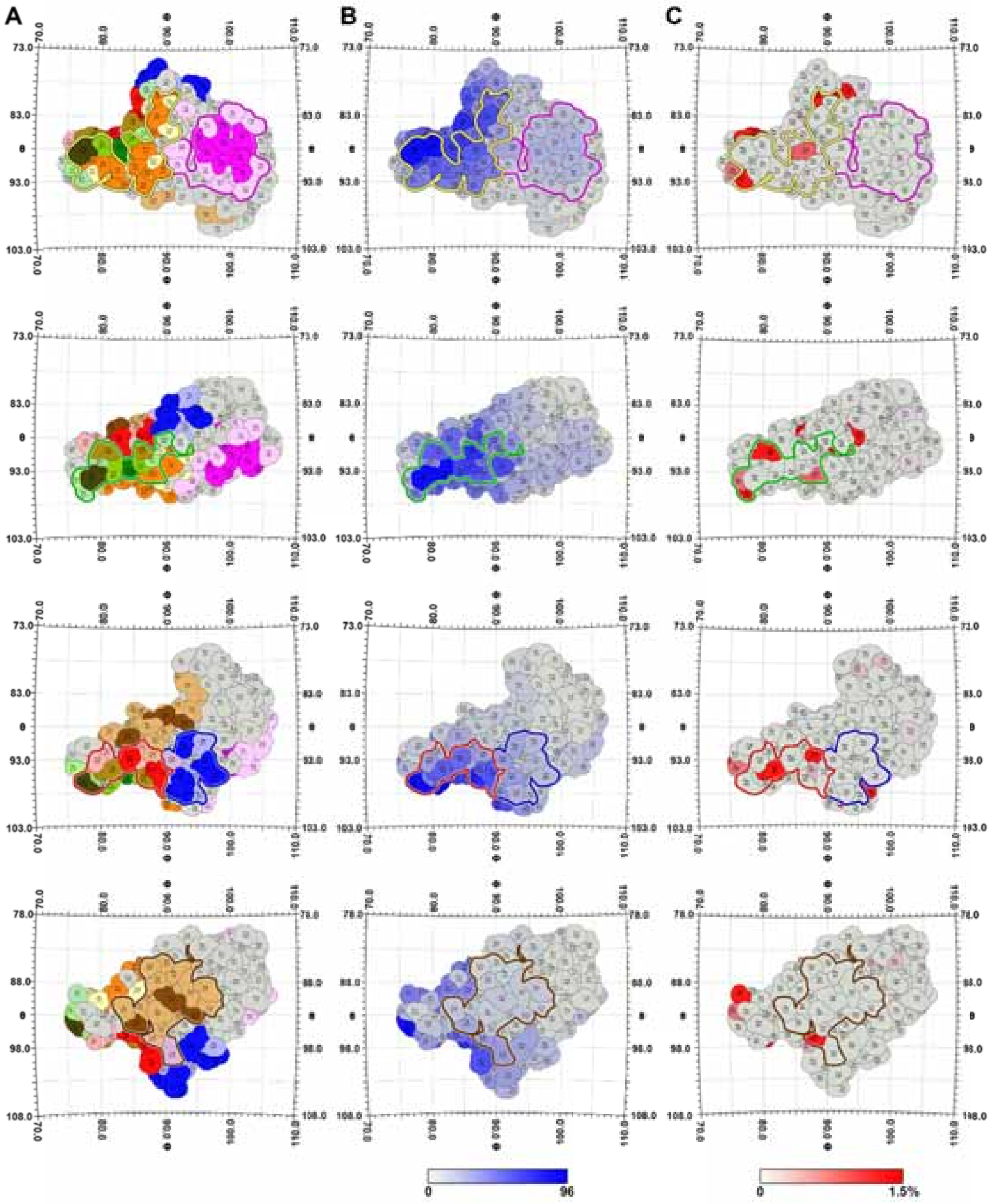

Fig. S13 Structural landscape of footprints on RBD surface.

A two-dimensional projection of the RBD surface was produced using RIVEM. Residues of RBD are colored in gray. (A) Residues of RBD belonging to antigenic patches (with targeting frequency >30%) recognized by six classes of NAbs are colored in the assigned color scheme (same to Fig. 2C), among which residues with "hot targeting frequency" (generally over 65%, but over 85% in class I) are shown in bright colors corresponding to the patches they belong to. Residues involved in

two (such as Y489, L452) or three (such as F486) antigenic patches are shown in a mixed color. (B) Heat map of antibody binding number. Residues are colored by the frequency of recognition by 171 Nabs. (C) Heat map for circulating variants with mutations on the RBD. Mutation frequency for each residue was calculated based on the datasets from GISAID.

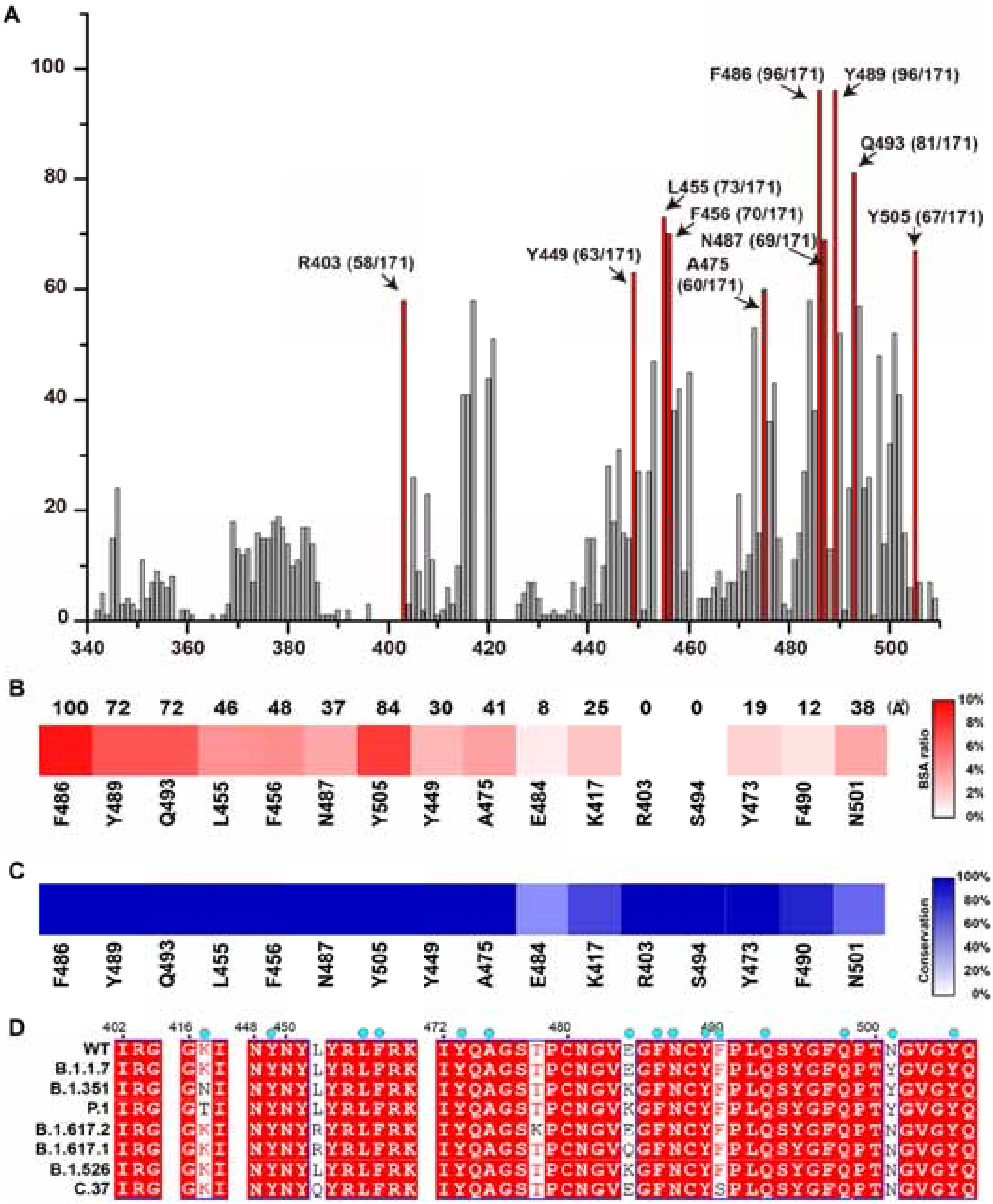

Fig. S14 Immunogenic analysis of the residues on SARS-CoV-2 RBD.

(A) Immunogenic analysis of the residues on SARS-CoV-2 RBD based on 171 Nabs. The vertical axis represents the number of Nabs that neutralize a certain site. Bars of the 10 hottest immunogenic residues in the histogram are filling with red. (B) Buried surface area (BSA) ratio upon binding to ACE2 of the 16 hottest immunogenic residues. BSA of these residues are listed above the chart. (C) Conservation of the 16 hottest immunogenic residues in eight SARS-CoV-2 strains (WT, B.1.1.7, B.1.351, P.1, B.1.617.2, B.1.617.1, B.1.526 and C.37). (D) Sequence alignment of SARS-CoV-2 WT and variant B.1.1.7, B.1.351, P.1, B.1.617.2, B.1.617.1, B.1.526 and C.37. The residues involved in direct interactions with ACE2 are marked with cyan balls.

A

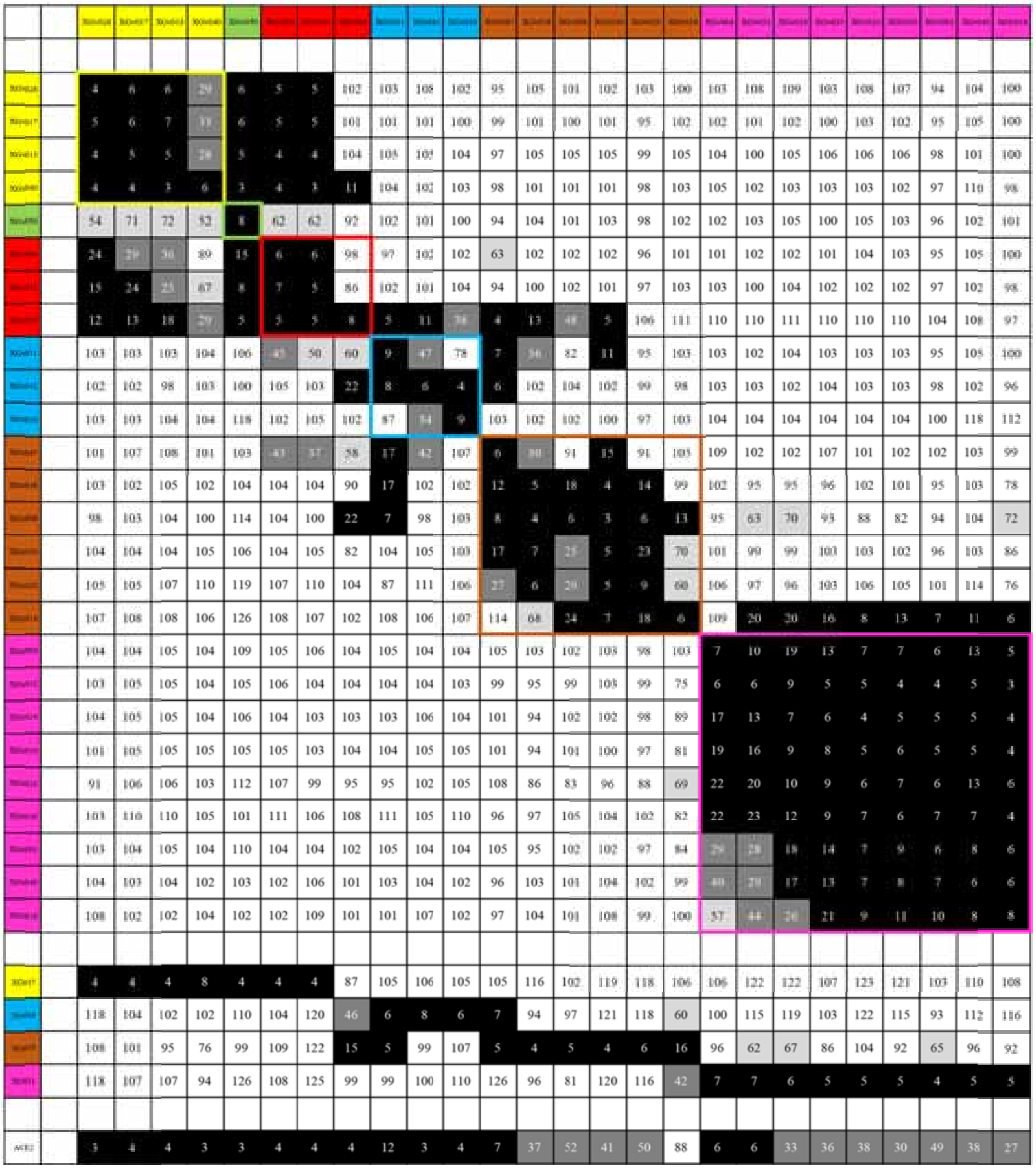

B

		XGv013	P4A1	XOm020	XG017	H4	414	[illegible]	[illegible]	F17	XG014	XOm016	XOm042	XG025	FC06	XOm058	XG011	A36-2	[illegible]	FC05
BD906		3	4	3	3	12	3	6	5	12	4	17	13	79	82	61	73	58	60	93
BD912		3	4	3	3	12	3	4	4	7	4	80	89	75	74	85	67	70	59	89
BD837		3	4	3	3	12	3	4	5	11	5	72	89	74	66	69	62	52	50	88
BD930		4	6	6	4	11	4	20	19	106	9	44	48	58	108	56	38	57	67	81
VA5-1		6	9	8	10	39	33	20	17	54	33	78	91	74	75	75	74	78	75	99
VA5-2		12	9	7	11	75	34	16	15	77	55	94	80	83	77	66	88	78	90	97
VA5-9		10	9	8	10	43	30	17	17	26	18	91	85	67	83	82	99	88	94	103
VA8-3		9	12	11	10	23	24	21	19	51	28	93	96	83	93	102	82	102	89	97
VA8-4		14	9	8	18	45	42	7	10	14	21	19	13	49	86	90	74	83	86	95
VA8-5		13	13	11	12	35	37	15	19	23	11	19	13	94	104	91	87	95	99	101
VA9-3		25	23	12	13	50	36	33	31	66	55	108	85	93	81	84	91	95	98	100
VA9-5		21	22	10	10	41	29	26	28	62	56	98	100	98	96	107	83	92	91	104
VA13-6		7	12	9	7	46	37	27	82	40	39	83	64	80	76	64	70	92	82	89
VA13-7		7	9	11	10	49	38	26	114	37	43	75	87	81	83	81	83	152	88	92
VA13-9		8	9	13	9	54	39	25	94	38	44	76	80	94	90	83	75	120	84	98
VA14-3		13	18	15	12	50	39	28	159	65	51	95	87	84	91	84	99	97	80	99
VA14-5		8	14	10	6	48	37	15	19	81	68	87	94	90	116	103	95	89	79	86
VA29-18		9	10	8	8	34	22	24	17	68	50	83	109	85	114	116	99	91	96	104
VA32-3		8	9	8	8	44	33	16	16	69	48	108	89	55	87	105	74	93	89	94
VA32-5		16	18	13	19	48	35	20	18	50	20	66	52	105	116	93	81	82	81	96
VA34-6		14	14	11	14	48	45	14	20	52	24	91	73	129	129	120	116	97	108	95
XG018		16	25	21	16	54	51	36	30	39	22	90	39	77	94	92	106	89	94	88
VA34-7		35	33	20	25	50	43	22	24	60	23	56	58	88	111	95	96	83	102	85
VA5-8		9	8	7	6	40	31	13	14	39	33	93	109	128	107	107	101	98	100	107
BD30-503		7	16	12	6	6	3	51	49	45	14	68	80	88	106	88	93	99	88	91
BD494		20	29	24	19	6	3	86	69	102	49	79	90	83	106	84	95	64	69	79
BD507		28	38	30	25	6	4	85	77	93	55	83	86	80	92	80	85	80	87	89
XG008		35	36	30	22	52	28	45	33	62	61	131	86	81	89	87	87	87	97	93
BD30-508		36	46	46	24	5	4	78	80	73	45	80	106	78	108	99	92	126	81	90
VA29-11		54	57	46	48	62	67	57	57	28	17	88	80	89	104	114	101	95	81	96

		XOv013	P4A1	XOv029	XO017	H8	414	[illegible]	[illegible]	P17	XO014	XOv018	XOv042	XO029	FC08	XOv030	XO011	A16-2	XG0004	FC05
BD836		3	3	3	3	12	3	4	3	6	4	56	67	72	67	63	51	71	32	93
BD674		5	6	15	5	11	4	23	16	42	5	70	37	49	50	72	45	33	46	78
BD790		31	24	26	25	12	3	75	57	76	20	46	52	95	85	82	79	50	82	100
BD915		19	36	28	28	10	5	67	51	68	43	73	55	43	66	76	58	82	51	82
BD913		49	48	57	63	12	3	86	70	86	26	42	60	10	53	76	73	69	78	93
BD748		45	63	56	42	10	4	53	68	96	12	54	25	5	17	46	45	50	37	81
BD693		39	56	49	44	12	4	54	62	99	25	81	36	55	64	70	53	59	51	80
BD30-315		7	15	11	8	6	3	41	44	63	35	77	41	64	103	73	86	99	81	93
BD30-613		27	30	30	27	5	4	82	89	80	43	78	72	66	118	91	100	106	84	97
BD30-616		9	18	14	9	6	3	45	52	64	24	94	87	75	108	67	82	100	80	93
VA29-13		20	22	17	11	31	14	31	33	46	18	27	27	73	103	89	104	99	85	105
BD504		16	22	16	12	6	3	71	54	72	31	100	89	78	91	84	93	85	91	105
BD618		6	12	11	8	9	3	45	33	85	31	85	91	78	103	94	93	93	94	97
VA29-12		55	57	48	63	32	61	57	54	70	64	53	54	81	113	94	91	78	78	101
XG003		95	86	92	78	46	23	69	81	108	39	74	47	107	95	77	91	83	101	83
XG004		74	74	91	83	21	28	117	52	46	9	99	64	143	140	42	67	55	80	94
XG009		49	48	39	36	18	36	57	31	44	60	71	59	119	102	81	94	91	94	96
XG043		44	47	48	41	15	50	47	40	42	44	50	43	74	87	72	76	97	82	99
BD498		54	51	54	37	6	5	127	89	97	67	80	88	71	119	95	87	86	88	87

		XGv013	P4A1	XGv028	XG017	H4	[illegible]	[illegible]	[illegible]	[illegible]	[illegible]	[illegible]	[illegible]	[illegible]	[illegible]	[illegible]	[illegible]	[illegible]	[illegible]	FC05
[illegible]		11	28	11	24	40	27	7	8	10	11	11	11	72	105	97	79	76	81	91
[illegible]		16	14	11	28	52	41	7	7	12	19	16	13	75	92	95	87	85	80	87
[illegible]		13	12	11	25	51	42	5	7	9	16	14	10	75	90	94	103	97	102	98
[illegible]		16	15	12	38	55	44	6	5	26	33	106	90	119	114	104	101	91	80	92
[illegible]		52	61	50	81	68	47	12	12	18	32	21	12	79	94	107	95	94	93	95
[illegible]		3	19	6	59	11	3	5	5	6	4	6	5	71	72	68	62	27	57	89
[illegible]		61	81	55	61	14	4	49	77	14	4	5	7	4	8	52	56	61	53	77
[illegible]		65	70	70	71	12	3	69	73	22	5	5	7	7	33	96	75	71	52	93
[illegible]		52	58	37	51	11	3	7	57	8	4	4	4	4	8	70	70	52	46	95
[illegible]		64	68	62	65	11	4	65	74	49	3	8	5	3	10	56	55	63	49	76
[illegible]		100	94	74	80	11	4	76	120	52	5	10	9	4	13	69	72	72	65	85
[illegible]		19	52	12	69	29	41	9	39	10	12	12	9	98	74	74	70	124	95	97
[illegible]		86	75	76	84	53	42	52	59	34	25	34	34	14	24	105	83	83	81	96
[illegible]		46	47	82	90	30	24	101	86	84	39	48	41	102	93	62	68	87	96	91
[illegible]		108	103	75	79	58	48	85	76	99	33	37	43	96	116	70	104	94	98	99
[illegible]		88	92	90	90	54	40	89	82	112	49	60	56	103	95	75	94	94	106	89
[illegible]		92	101	86	99	57	26	90	87	103	30	54	46	105	98	83	87	102	103	94
[illegible]		102	93	101	84	109	98	101	94	102	92	98	116	100	80	86	98	110	112	106
[illegible]		93	100	73	98	76	90	81	79	161	41	75	62	78	74	7	12	5	7	84
[illegible]		44	66	101	70	54	72	66	89	80	87	63	96	77	66	68	39	22	29	89
[illegible]		56	82	55	84	67	75	76	99									21	22	89
[illegible]		95	82	60	117	66	76	81	114	129	82	113	125	52	127	79	80	67	70	81
[illegible]		82	93	88	91	136	104	91	90	104	99	102	108	83	102	57	22	21	26	97
[illegible]		70	73	86	84	62	68	72	82	79	79	76	71	78	69	40	35	29	44	88

Fig. S15 Data sheets of ELISA assay of NAbs neutralizing SARS-CoV-2 RBD. Data of ELISA assay of different NAbs are listed in (A) and (B). NAbs corresponding to Class I-VI are filled with yellow, green, red, blue, brown and magenta colors, respectively. Values in the tables are filled with black (<25), grey (25~50), silver (50~70) and white (>70).

Table S1. Information of recombinant antibodies

Antibody ID	IGH V gene	SHM	Binding Affinity - K_D (nM)				
			WT	B.1.1.7	P.1	B.1.351	B.1.617.2
XGv001	IGHV3-21	5.08%	0.17	0.23	0.18	0.24	0.30
XGv002	IGHV3-33	6.76%	4.15	4.42	7.18	5.80	3.91
XGv003	IGHV1-69	7.12%	0.10	0.21	NS	NS	0.71
XGv004	IGHV1-18	3.41%	0.14	0.10	0.11	0.16	0.20
XGv005	IGHV1-69-2	7.48%	3.17	3.69	28.03	1.25	7.94
XGv006	IGHV1-18	8.53%	3.68	3.06	9.33	6.65	3.86
XGv007	IGHV4-61	3.01%	1.70	4.22	5.34	5.82	4.50
XGv008	IGHV4-39	2.68%	0.47	0.45	0.30	0.34	0.28
XGv009	IGHV3-30	4.05%	1.30	9.85	9.63	NS	22.60
XGv010	IGHV3-74	0.68%	1.83	0.52	NS	NS	33.50
XGv011	IGHV3-30	6.94%	0.10	0.50	0.64	3.93	0.66
XGv012	IGHV4-39	6.10%	0.25	4.51	2.90	3.10	1.16
XGv013	IGHV3-53	3.75%	0.26	1.22	5.58	9.74	0.29
XGv014	IGHV3-9	4.76%	0.37	0.40	0.45	0.26	0.39
XGv015	IGHV3-30	4.73%	21.01	19.93	20.39	31.41	21.67
XGv016	IGHV1-46	8.14%	0.16	0.26	0.17	0.34	0.34
XGv017	IGHV3-53	5.12%	0.31	0.31	1.21	8.43	0.26
XGv018	IGHV3-33	3.38%	0.21	0.62	1.67	0.79	0.89
XGv019	IGHV3-13	6.85%	0.59	0.24	0.84	0.43	0.48
XGv020	IGHV3-30	4.41%	0.29	0.67	0.19	0.24	0.17
XGv021	IGHV3-30	5.41%	0.26	0.35	0.40	0.18	0.41
XGv022	IGHV3-30	4.78%	25.60	27.40	13.70	15.80	20.70
XGv023	IGHV3-13	5.48%	0.21	0.62	1.67	0.79	0.89
XGv024	IGHV3-13	2.05%	0.76	0.51	1.08	0.67	0.58
XGv025	IGHV3-30-3	4.05%	1.22	2.20	3.74	4.46	3.02
XGv026	IGHV3-53	6.83%	0.23	0.33	0.69	0.81	0.15
XGv027	IGHV4-34	7.67%	4.16	7.78	6.20	13.73	6.64
XGv028	IGHV2-5	5.03%	0.68	1.11	NS	1.78	NS
XGv029	IGHV7-4-1	2.70%	14.00	13.60	16.10	13.50	17.30
XGv030	IGHV3-48	3.73%	1.30	1.83	2.12	1.85	1.68
XGv031	IGHV1-69	2.72%	0.26	0.16	0.50	0.47	0.48
XGv032	IGHV3-13	5.14%	0.16	0.16	0.11	0.09	0.09
XGv033	IGHV4-4	5.48%	1.86	2.43	4.10	4.40	3.24
XGv034	IGHV3-48	5.74%	4.13	7.19	9.17	11.63	6.42
XGv035	IGHV7-4-1	3.38%	3.98	3.76	5.01	3.45	5.22
XGv036	IGHV1-18	8.50%	0.63	0.99	1.82	1.51	1.69
XGv038	IGHV3-33	3.04%	0.43	0.59	0.33	0.28	0.29
XGv039	IGHV3-13	5.82%	1.00	0.75	1.36	1.54	0.66
XGv040	IGHV3-53	2.73%	0.91	0.68	11.56	18.03	1.10
XGv041	IGHV3-30-3	4.08%	NS	NS	NS	NS	NS
XGv042	IGHV2-5	4.71%	0.16	0.17	0.29	0.16	0.27
XGv043	IGHV4-39	7.59%	9.79	NS	6.39	NS	NS
XGv044	IGHV3-23	6.10%	2.17	13.93	17.71	23.15	22.84
XGv045	IGHV4-61	5.74%	0.40	0.94	1.29	1.41	0.92
XGv046	IGHV4-34	9.31%	0.53	1.10	0.95	2.03	1.24
XGv047	IGHV1-69	5.86%	0.10	0.08	0.17	0.32	0.79
XGv049	IGHV3-13	6.48%	0.39	0.17	0.58	0.40	0.18
XGv050	IGHV3-13	4.79%	0.26	0.19	0.37	0.60	2.87
XG011	IGHV3-13	4.11%	2.74	1.74	3.40	2.50	2.19
XG014	IGHV5-51	2.36%	1.78	1.13	1.90	1.30	1.14
XG017	IGHV3-66	2.40%	1.52	1.28	NS	NS	0.75
XG025	IGHV3-33	3.05%	1.60	0.77	1.36	1.41	0.64
H014	N/A	N/A	0.11	0.25	0.21	0.26	0.14
HB27	N/A	N/A	0.99	1.88	1.32	1.57	1.37
FC08	IGHV1-69	0.33%	0.10	0.25	0.46	0.49	1.32
P17	N/A	N/A	3.97	9.66	NS	NS	25.00
414	IGHV1-46	0.00%	0.92	NS	NS	NS	5.99
A8-1	IGHV3-53	1.71%	0.51	0.86	2.45	12.62	0.34
S309	IGHV1-18	2.78%	0.75	0.55	1.01	0.93	0.60
REGN10987	IGHV3-30	1.37%	1.91	3.14	2.84	3.43	4.16
553	IGHV3-7	3.12%	3.06	9.07	7.49	8.93	10.48
1-57	IGHV3-72	0.70%	5.86	16.22	23.82	24.04	NS
A34-2	IGHV3-9	3.09%	0.36	0.31	2.08	1.45	0.41
A5-10	IGHV3-23	2.04%	1.78	1.96	1.43	1.99	0.33
B38	IGHV3-53	1.00%	21.89	14.03	NS	NS	25.80
BD-368-2	IGHV3-23	7.98%	2.62	6.67	NS	NS	31.60
C121	IGHV1-2	1.02%	1.49	4.39	NS	NS	1.98
C135	IGHV3-30	2.05%	5.96	15.52	17.09	21.20	8.72
C144	IGHV3-53	1.37%	21.87	62.99	NS	NS	51.93
CB6	IGHV3-66	1.00%	17.25	51.64	NS	NS	46.18
CC12.3	IGHV3-53	N/A	7.70	21.26	NS	NS	12.14
COVA1-16	IGHV1-46	0.34%	1.02	0.95	4.01	2.10	8.71
COVA2-04	IGHV4-4	1.10%	33.13	30.49	NS	NS	29.48

COVA2-39	IGHV3-53	1.10%	2.69	7.62	NS	NS	3.62
CV07-270	IGHV3-11	0.68%	22.87	40.06	NS	NS	NS
DH1047	IGHV1-46	N/A	0.21	0.60	0.80	0.73	0.49
CC12.1	IGHV3-53	N/A	8.85	-	-	-	9.76
COVOX-150	IGHV3-53	3.16%	1.40	-	-	-	1.79
COVOX-222	IGHV3-53	2.46%	1.22	-	-	-	1.48
COVOX-269	IGHV3-53	4.21%	3.88	-	-	-	4.64
COVOX-40	IGHV3-53	1.75%	0.89	-	-	-	1.12
P2C-1F11	IGHV3-11	1.75%	9.75	-	-	-	10.87
BD-236	IGHV3-53	3.10%	15.80	-	-	-	-
BD-604	IGHV3-53	4.00%	0.31	-	-	-	-
BD-623	IGHV3-66	2.75%	1.30	-	-	-	-
BD-629	IGHV3-53	1.70%	0.93	-	-	-	-
BD23	IGHV7-4-1	0.00%	34.99	-	-	-	-
BD30-494	IGHV3-53	1.02%	7.04	-	-	-	-
BD30-498	IGHV3-53	1.71%	16.37	-	-	-	-
BD30-503	IGHV3-53	2.39%	1.81	-	-	-	-
BD30-504	IGHV1-NL1	7.67%	4.32	-	-	-	-
BD30-505	IGHV3-53	N/A	4.01	-	-	-	-
BD30-507	IGHV3-53	1.71%	2.82	-	-	-	-
BD30-508	IGHV3-53	1.71%	10.65	-	-	-	-
BD30-515	IGHV3-66	2.05%	1.36	-	-	-	-
BD30-605	IGHV3-53	0.40%	15.12	-	-	-	-
BD30-613	IGHV3-66	0.00%	6.88	-	-	-	-
BD30-616	IGHV3-66	1.03%	3.48	-	-	-	-
BD30-618	IGHV3-66	4.79%	1.00	-	-	-	-
BD667	IGHV3-11	7.12%	2.56	-	-	-	-
BD674	IGHV4-4	4.39%	0.34	-	-	-	-
BD692	IGHV3-48	1.01%	0.29	-	-	-	-
BD693	IGHV3-15	2.68%	0.22	-	-	-	-
BD702	IGHV3-48	2.38%	0.85	-	-	-	-
BD744	IGHV3-9	4.05%	0.21	-	-	-	-
BD748	IGHV3-48	4.41%	0.73	-	-	-	-
BD771	IGHV3-15	4.65%	0.21	-	-	-	-
BD790	IGHV1-2	4.75%	0.75	-	-	-	-
BD804	IGHV3-21	3.04%	0.37	-	-	-	-
BD812	IGHV5-51	5.90%	0.32	-	-	-	-
BD813	IGHV3-53	3.44%	0.42	-	-	-	-
BD826	IGHV4-4	7.46%	0.04	-	-	-	-
BD836	IGHV1-58	6.14%	0.09	-	-	-	-
BD837	IGHV1-58	5.80%	0.07	-	-	-	-
BD868	IGHV3-53	7.85%	0.24	-	-	-	-
BD870	IGHV3-48	9.15%	0.52	-	-	-	-
BD897	IGHV1-69	9.15%	0.11	-	-	-	-
BD901	IGHV1-69	10.14%	0.12	-	-	-	-
BD902	IGHV3-33	5.07%	0.74	-	-	-	-
BD906	IGHV4-39	9.70%	0.25	-	-	-	-
BD907	IGHV7-4-1	4.05%	0.39	-	-	-	-
BD912	IGHV3-21	6.25%	0.50	-	-	-	-
BD913	IGHV1-69	1.69%	0.71	-	-	-	-
BD914	IGHV1-69	4.05%	0.14	-	-	-	-
BD915	IGHV4-4	2.41%	0.53	-	-	-	-
BD930	IGHV4-39	3.68%	0.17	-	-	-	-
BD941	IGHV4-31	4.70%	0.28	-	-	-	-
BD977	IGHV3-30	4.73%	0.13	-	-	-	-
BG1-22	IGHV3-66	N/A	21.79	-	-	-	-
C002	IGHV3-30	0.34%	19.66	-	-	-	-
C102	IGHV3-53	0.68%	14.27	-	-	-	-
C105	IGHV3-53	0.34%	23.72	-	-	-	-
C110	IGHV5-51	0.34%	0.90	-	-	-	-
C119	IGHV1-46	0.34%	5.04	-	-	-	-
C1A-B12	IGHV3-53	1.40%	3.89	-	-	-	-
C1A-B3	IGHV3-53	2.11%	16.96	-	-	-	-
C1A-C2	IGHV3-53	1.40%	15.39	-	-	-	-
C1A-F10	IGHV3-53	2.11%	17.69	-	-	-	-
COVOX-45	IGHV1-33	1.39%	5.25	-	-	-	-
COVOX-75	IGHV3-30	4.86%	17.06	-	-	-	-
CV30	IGHV3-53	N/A	11.31	-	-	-	-
H4	IGHV1-2	0.00%	8.56	-	-	-	-
LY-CoV481	IGHV3-53	N/A	7.76	-	-	-	-
LY-CoV488	IGHV3-53	N/A	35.83	-	-	-	-
P2B-1A10	IGHV3-53	0.35%	29.84	-	-	-	-
P4A1	IGHV3-53	N/A	13.58	-	-	-	-
P5A-1B8	IGHV3-53	1.40%	10.57	-	-	-	-
P5A-2G9	IGHV3-33	0.00%	7.52	-	-	-	-
P5A-3A1	IGHV3-53	0.00%	11.28	-	-	-	-
REGN10933	IGHV3-11	1.35%	1.39	-	-	-	-
S2A4	IGHV3-7	N/A	5.60	-	-	-	-
S304	IGHV3-13	2.11%	1.99	-	-	-	-

VA11-1_H	IGHV3-30	1.36%	0.52	-	-	-	-
VA13-6_H	IGHV3-66	0.34%	0.82	-	-	-	-
VA13-7_H	IGHV3-66	0.34%	0.98	-	-	-	-
VA13-8_H	IGHV1-69	2.71%	0.21	-	-	-	-
VA13-9_H	IGHV3-66	0.34%	1.14	-	-	-	-
VA14-3_H	IGHV3-66	1.37%	1.28	-	-	-	-
VA14-5_H	IGHV3-53	3.41%	1.10	-	-	-	-
VA29-10-A_H	IGHV1-69	3.07%	0.42	-	-	-	-
VA29-11_H	IGHV3-53	1.73%	1.75	-	-	-	-
VA29-12_H	IGHV1-69	3.05%	4.94	-	-	-	-
VA29-13_H	IGHV2-5	1.00%	2.80	-	-	-	-
VA29-14_H	IGHV1-69	5.14%	0.40	-	-	-	-
VA29-18_H	IGHV3-53	2.73%	0.99	-	-	-	-
VA29-5_H	IGHV1-69	3.04%	4.22	-	-	-	-
VA29-6_H	IGHV1-69	3.08%	0.35	-	-	-	-
VA32-1_H	IGHV3-66	4.78%	1.28	-	-	-	-
VA32-5_H	IGHV1-69	3.05%	1.02	-	-	-	-
VA34-4_H	IGHV3-53	2.08%	0.32	-	-	-	-
VA34-6_H	IGHV4-34	3.41%	1.04	-	-	-	-
VA34-7_H	IGHV4-34	3.41%	0.97	-	-	-	-
VA5-1_H	IGHV3-66	1.71%	3.87	-	-	-	-
VA5-2_H	IGHV3-66	1.71%	0.58	-	-	-	-
VA5-8_H	IGHV3-53	3.07%	5.49	-	-	-	-
VA5-9_H	IGHV3-66	1.37%	1.15	-	-	-	-
VA8-3_H	IGHV3-66	4.10%	0.48	-	-	-	-
VA8-4_H	IGHV3-49	3.32%	4.30	-	-	-	-
VA8-5_H	IGHV3-49	4.32%	3.68	-	-	-	-
VA9-3_H	IGHV3-53	1.71%	1.21	-	-	-	-
VA9-5_H	IGHV3-53	3.07%	2.33	-	-	-	-
XG003	IGHV2-70	5.69%	2.29	-	-	-	-
XG004	IGHV2-70	0.00%	1.19	-	-	-	-
XG005	IGHV2-5	1.69%	0.67	-	-	-	-
XG008	IGHV1-69	2.36%	0.41	-	-	-	-
XG009	IGHV1-8	1.01%	25.28	-	-	-	-
XG013	IGHV2-70	2.68%	0.50	-	-	-	-
XG016	IGHV2-5	4.38%	2.35	-	-	-	-
XG031	IGHV2-5	3.68%	1.24	-	-	-	-
XG036	IGHV1-69	5.78%	1.71	-	-	-	-
XG038	IGHV1-18	4.39%	1.42	-	-	-	-
XG043	IGHV3-20	0.34%	7.39	-	-	-	-

Table S2. Neutralizing titers of recombinant antibodies

Antibody ID	Neutralizing Titer - IC_{50} (ng/mL)									
	Pseudovirus						Authentic virus			
	WT	B.1.1.7	P.1	B.1.351	B.1.617.2	SARS-CoV	WT	P.1	B.1.351	B.1.617.2
XGv001	736	519	458	792	815	115	16667	16667	16667	12500
XGv002	1201	>2000	>2000	>2000	>2000	>2000	8333	>50000	>50000	>50000
XGv003	5	7	>2000	>2000	20	>2000	24	>50000	>50000	33
XGv004	118	131	30	72	144	>2000	391	1042	1563	1042
XGv005	>2000	>2000	>2000	>2000	>2000	>2000	>50000	>50000	>50000	>50000
XGv006	1563	>2000	>2000	>2000	>2000	>2000	2083	>50000	>50000	>50000
XGv007	>2000	>2000	>2000	>2000	>2000	>2000	50000	>50000	>50000	>50000
XGv008	1073	955	1436	1769	2000	>2000	1042	>50000	>50000	>50000
XGv009	>2000	>2000	>2000	>2000	>2000	>2000	50000	>50000	>50000	>50000
XGv010	6	6	>2000	>2000	>2000	>2000	24	>50000	>50000	>50000
XGv011	25	37	56	178	9	>2000	1042	2083	4167	391
XGv012	2000	>2000	>2000	>2000	>2000	>2000	33333	>50000	>50000	>50000
XGv013	10	10	2	5	12	>2000	65	130	195	130
XGv014	1199	745	414	1021	1022	486	33333	50000	33333	8333
XGv015	1406	1421	291	590	992	>2000	12500	6250	33333	1563
XGv016	1	1	1	2	4	>2000	24	24	24	24
XGv017	16	13	4	6	16	>2000	98	130	130	195
XGv018	839	618	389	449	756	73	4167	12500	12500	8333
XGv019	847	893	801	747	1104	>2000	12500	12500	16667	16667
XGv020	723	1132	1365	1531	1459	>2000	6250	33333	33333	33333
XGv021	>2000	>2000	>2000	>2000	>2000	>2000	>50000	>50000	>50000	>50000
XGv022	>2000	>2000	>2000	>2000	>2000	>2000	>50000	>50000	>50000	>50000
XGv023	1126	961	959	614	957	741	4167	16667	12500	16667
XGv024	603	702	955	1028	1459	>2000	4167	16667	8333	16667
XGv025	2000	>2000	>2000	>2000	>2000	>2000	12500	>50000	>50000	>50000
XGv026	10	13	3	5	9	>2000	33	24	24	98
XGv027	1259	2083	2083	2083	2083	>2000	49	>50000	>50000	1563
XGv028	2000	>2000	>2000	>2000	>2000	>2000	16667	>50000	>50000	50000
XGv029	2000	>2000	>2000	>2000	>2000	>2000	33333	>50000	>50000	>50000
XGv030	850	809	865	1125	1101	41	24	24	33	33333
XGv031	4	5	1	2	11	>2000	24	24	24	24
XGv032	194	350	243	348	439	>2000	1563	4167	4167	2083
XGv033	>2000	>2000	>2000	>2000	>2000	>2000	>50000	>50000	>50000	>50000
XGv034	>2000	>2000	>2000	>2000	>2000	>2000	>50000	>50000	>50000	>50000
XGv035	2000	>2000	>2000	>2000	>2000	>2000	6250	>50000	>50000	>50000
XGv036	2000	>2000	>2000	>2000	>2000	>2000	12500	>50000	>50000	>50000
XGv038	283	220	334	764	833	>2000	3125	8333	12500	>50000
XGv039	1165	1695	690	958	1317	1250	195	391	2083	12500
XGv040	21	40	6	15	16	>2000	130	391	391	521
XGv041	>2000	>2000	>2000	>2000	>2000	>2000	>50000	>50000	>50000	>50000
XGv042	2	2	1	2	5	>2000	24	24	24	33
XGv043	181	>2000	>2000	2083	>2000	>2000	781	>50000	>50000	>50000
XGv044	>2000	>2000	>2000	>2000	>2000	>2000	>50000	>50000	>50000	>50000
XGv045	2000	>2000	>2000	>2000	>2000	>2000	25000	>50000	>50000	>50000
XGv046	586	1447	1158	1159	2000	>2000	8333	8333	6250	>50000
XGv047	20	12	4	9	56	>2000	6250	16667	16667	781
XGv049	911	937	578	634	1168	552	65	195	195	12500
XGv050	5	5	6	3	3	>2000	391	2083	1042	98
XG011	1832	1735	1236	1145	1614		-	-	-	-
XG014	2	2	2	3	3		-	-	-	-
XG017	9	10	1966	1248	16		-	-	-	-
XG025	163	442	255	640	820		-	-	-	-
H014	86	212	152	246	143		-	-	-	-
HB27	1	309	48	130	1		-	-	-	-
FC08	10	5	6	28	1207		-	-	-	-
P17	3	6	>2000	>2000	13		-	-	-	-
414	18	>2000	>2000	>2000	1124		-	-	-	-
A8-1	6	4	4	7	5		-	-	-	-
S309	30	23	12	7	12		-	-	-	-
REGN10987	2	1	1	1	3		-	-	-	-

Table S3. Data collection, processing and refinement

Protein	Spike in complex with XGv013	Spike in complex with XGv043	Spike in complex with XGv004, XGv016 and XGv030	Spike in complex with XGv026 and XGv046	Spike in complex with XGv018, XGv038 and XGv042
Voltage (kV)	300	300	300	300	300
Detector	K2	K2	K2	K2	K2
Pixel size (Å)	1.04	1.04	1.04	1.04	1.04
Electron dose (e^-/Å^2)	60	60	60	60	60
Defocus range (μm)	1.5-2.7	1.5-2.7	1.5-2.7	1.5-2.7	1.5-2.7
Final particles	21,329	13,548	20,738	9,617	13,637
Final resolution (Å)	5.2	6.5	7.0	7.8	6.7
